# Adenosine Signaling and Clathrin-Mediated Endocytosis of Glutamate AMPA Receptors in Delayed Hypoxic Injury in Rat Hippocampus: Role of Casein Kinase 2

**DOI:** 10.1007/s12035-020-02246-0

**Published:** 2021-01-07

**Authors:** Xin Qin, Michael G. Zaki, Zhicheng Chen, Elisabet Jakova, Zhi Ming, Francisco S. Cayabyab

**Affiliations:** 1 Department of Surgery, Neuroscience Research Cluster, College of Medicine, University of Saskatchewan, Room GD30.4, D-Wing Health Science Building, 107 Wiggins Road, Saskatoon, SK S7N 5E5 Canada; 2 Department of Pharmacology and Toxicology, Faculty of Pharmacy, Kafrelsheikh University, Kafrelsheikh, Egypt

**Keywords:** Adenosine receptors, AMPA receptors, Casein kinase 2, Ischemic injury, Synaptic plasticity, Perampanel, Clathrin-mediated endocytosis, Hippocampus, Synaptic transmission, Dynamin

## Abstract

Chronic adenosine A1R stimulation in hypoxia leads to persistent hippocampal synaptic depression, while unopposed adenosine A2AR receptor stimulation during hypoxia/reperfusion triggers adenosine-induced post-hypoxia synaptic potentiation (APSP) and increased neuronal death. Still, the mechanisms responsible for this adenosine-mediated neuronal damage following hypoxia need to be fully elucidated. We tested the hypothesis that A1R and A2AR regulation by protein kinase casein kinase 2 (CK2) and clathrin-dependent endocytosis of AMPARs both contribute to APSPs and neuronal damage. The APSPs following a 20-min hypoxia recorded from CA1 layer of rat hippocampal slices were abolished by A1R and A2AR antagonists and by broad-spectrum AMPAR antagonists. The inhibitor of GluA2 clathrin-mediated endocytosis Tat-GluA2-3Y peptide and the dynamin-dependent endocytosis inhibitor dynasore both significantly inhibited APSPs. The CK2 antagonist DRB also inhibited APSPs and, like hypoxic treatment, caused opposite regulation of A1R and A2AR surface expression. APSPs were abolished when calcium-permeable AMPAR (CP-AMPAR) antagonist (IEM or philanthotoxin) or non-competitive AMPAR antagonist perampanel was applied 5 min after hypoxia. In contrast, perampanel, but not CP-AMPAR antagonists, abolished APSPs when applied during hypoxia/reperfusion. To test for neuronal viability after hypoxia, propidium iodide staining revealed significant neuroprotection of hippocampal CA1 pyramidal neurons when pretreated with Tat-GluA2-3Y peptide, CK2 inhibitors, dynamin inhibitor, CP-AMPAR antagonists (applied 5 min after hypoxia), and perampanel (either at 5 min hypoxia onset or during APSP). These results suggest that the A1R-CK2-A2AR signaling pathway in hypoxia/reperfusion injury model mediates increased hippocampal synaptic transmission and neuronal damage via calcium-permeable AMPARs that can be targeted by perampanel for neuroprotective stroke therapy.

**Supplementary Information:**

The online version contains supplementary material available at 10.1007/s12035-020-02246-0.

## Introduction

Ischemic stroke occurs when tissue perfusion is reduced to part of the CNS, which results in hypoxic conditions and can result in permanent damage to brain parenchyma [1]. Ischemia/hypoxia in the brain can cause short-term cognitive and memory deficits [2], but the long-term effects of hypoxia are more widespread and includes permanent brain damage that can lead to disorders such as post-stroke dementia and depression [3]. Adenosine, an endogenous neuromodulator, is known to play a critical role in the brain’s response to neurotoxic insults such as hypoxia, in part by modulating synaptic transmission, although the mechanism by which this occurs is not fully elucidated. The inhibitory adenosine A1 receptor (A1R) and the excitatory adenosine A2A receptor (A2AR) are both involved in response to elevated extracellular adenosine during hypoxic/ischemic injury, and their activation is thought to be either neuroprotective (A1R) or neurodegenerative (A2AR) [4, 5]. Thus, it has been suggested that either A1R agonism or A2AR antagonism could be neuroprotective in ischemia/hypoxia, and indeed there have been many adenosine-based therapeutics tested with varying success [4, 6–8].

The neuroprotective effect of A1R is short lived because of desensitization and internalization of A1Rs occurring following their prolonged activation [9, 10]. In addition, we previously reported that hippocampal A1Rs and AMPARs are downregulated, whereas A2ARs are upregulated following a 20-min hypoxic insult or using a small vessel focal cortical ischemia animal model [11, 12]. We also found physical co-localization between both GluA1 and GluA2 subunits of AMPARs with A1R, but not between A2ARs and AMPARs, which suggests a functional interaction between A1Rs and AMPARs [11]. During hypoxia, a significant reduction in synaptic transmission occurs [13–15], along with decreased cell surface expression of GluA1 and GluA2 AMPAR subunits after a 20-min hypoxic insult [12], which are both thought to be neuroprotective mechanisms during neuronal insult. In addition, clathrin-mediated endocytosis of both A1Rs and GluA2 and GluA1 AMPARs have been observed to occur during prolonged A1R stimulation or in a 20-min hypoxic insult [11, 12, 16]. If normoxic conditions were reintroduced following hypoxia, we found increased surface expression of GluA1-containing AMPARs, whereas GluA2-containing AMPARs remained depressed [12], which is important because GluA2-lacking AMPARs are permeable to Ca^2+^ and can lead to excitotoxicity [17]. This post-hypoxia elevation of Ca^2+^-permeable AMPAR (CP-AMPAR) leads to increased synaptic excitability [17–19], a phenomenon that we have termed adenosine-induced post-hypoxia synaptic potentiation (APSP) [12]. However, the cellular basis for this enhanced synaptic potentiation and neuronal damage remains to be fully elucidated.

Previous studies showed that the protein kinase casein kinase 2 (CK2) activity declined in hippocampal CA1 region and striatum after brief ischemic insult and caused neuronal damage, whereas CK2 activity increased in brain regions that were resistant to ischemic damage [20, 21]. CK2 activity increases the desensitization rates of Gα_s_-coupled G protein coupled receptors (GPCRs), such as A2ARs, dopamine D1 receptor, serotonin 5-HT4 receptor, and M3-muscarinic receptor [22–26], which results in decreased surface expression of Gα_s_-coupled GPCRs. Furthermore, we previously observed increased A2ARs in our in vivo stroke model [11] and also reported increased surface GluA1 subunits but persistently depressed surface GluA2 subunits during post-hypoxia normoxic reperfusion [12]. Moreover, a previous study reported that protein phosphatase 2A (PP2A) activation can be prevented by inhibiting CK2-PP2A interaction [27]. Since PP2A activation after A1R stimulation was previously shown to decrease the surface expression of pSer845-GluA1 AMPARs [12], we sought to examine the role of CK2 in adenosine receptor signaling and hippocampal neuronal damage in hypoxic injury. In particular, we determined whether CK2 reciprocally regulates A1R and A2AR surface expression, which in turn leads to increased APSPs during hypoxia/reperfusion injury in hippocampal brain slices.

In addition to targeting adenosine receptors directly, modulating their downstream targets has been suggested as a possible therapeutic strategy [4, 28]. The present study aims to examine adenosine-mediated glutamatergic AMPA receptor (AMPAR) and NMDA receptor (NMDAR) regulation in hypoxia and its role in hippocampal synaptic transmission and neuronal damage. Therapeutic targeting of these glutamatergic receptors has been explored in various animal stroke models [29]. This study compares the effects of the broad-spectrum AMPAR antagonists CNQX, DNQX, and NBQX, and the selective CP-AMPAR antagonists Philanthotoxin-74 and IEM 1460, with the NMDAR antagonists MK-801 and D-APV using a 20-min hypoxic insult. Although both AMPARs and NMDARs have been suggested as therapeutic targets in cerebral ischemia, the abovementioned drugs are not ideal for clinical applications [30–32]. Recently, perampanel (Fycompa®), a non-competitive AMPAR antagonist, was developed and has already been FDA-approved for anti-seizure therapy [33–36]. In this study, we further clarified the roles of AMPARs, NMDARs, and CP-AMPARs in hypoxia-induced changes in synaptic transmission and neuronal damage and explored perampanel’s potential for neuroprotective stroke therapy.

## Methods

### Animal Subjects

This work was approved by the University of Saskatchewan’s Animal Research Ethics Board and adhered to the Canadian Council on Animal Care guidelines for humane animal use (Approved Animal Use Protocol Number: 20070090). The following experimental design, analysis and reported number of research animals used also complied with the ARRIVE guidelines for reporting experiments involving animal use to ensure all efforts were made to minimize animal suffering and the number of animals used in this study [37]. Male Sprague-Dawley rats at 20–30 days old (Charles River Canada, Montreal, Canada) were used in all studies. Rats were housed two per cage in standard polypropylene cages in a temperature controlled (21 °C) colony room on a 12/12-h light/dark cycle with free access to food and water. Experimental procedures were carried out during the light phase. We studied male Sprague-Dawley rats in the current study to continue and further interrogate similar mechanisms elucidated in our previous studies that used male rats alone [38].

### Preparation of Acute Hippocampal Slices

Rats were first anesthetized with halothane and were rapidly decapitated. The brains were then immediately transferred into ice-cold oxygenated high sucrose dissection solution. The solution was oxygenated by continuously aerating the solution with 95% O_2_/5% CO_2_. The recipe for the high sucrose dissection solution is as follows (in mM): 87 NaCl, 7.0 MgCl_2_, 1.25 NaH_2_PO_4_, 1.25 NaH_2_PO_4_, 25 glucose, 25 NaHCO_3_, 2.5 KCl, and 0.5 CaCl_2_ [11, 38]. Acute hippocampal slices were taken at 400 μm thickness using a Leica VTS1200S vibratome (Leica Instruments, Germany) while fully submerged in the same ice-cold dissection solution as above. Hippocampal slices were immediately transferred into oxygenated artificial cerebrospinal fluid (aCSF) at room temperature. The recipe for aCSF is described as follows (in mM): 2.5 KCl, 126 NaCl, 26 NaHCO_3_, 2.0 MgCl_2_, 10 glucose, 1.25 NaH_2_PO_4_, and 2.0 CaCl_2_ [11]. Prior to use, slices were allowed to equilibrate by incubating in oxygenated aCSF at room temperature for at least 1 h following slicing.

### Hippocampal Lysate Preparation, Biotinylation, and Western Blotting

Following the hippocampal tissue preincubation with the various treatments (see below), hippocampal slices were treated with NHS-SS-Biotin (1 mg/mL, Thermo Scientific) for 1 h at 4 °C to isolate surface proteins as described before [11, 12, 16]. The biotin reaction was quenched with glycine buffer containing 192 mM glycine and 25 mM Tris (pH 8.3). Slices were then transferred to homogenization tubes and homogenized in lysis buffer (pH 8.0) containing 50 mM Tris, 150 mM NaCl, 1 mM EDTA, 1 mM NaF, and the following protease inhibitors: 1 mM PMSF, 10 µg/mL aprotinin, 10 µg/mL pepstatin A, 10 µg/mL leupeptin, 2 mM Na_3_VO_4_, 20 mM sodium pyrophosphate, 3 mM benzamidine hydrochloride, and 4 mM glycerol 2-phosphate with 1% NP-40 detergent.

A Bradford assay was performed with DC protein assay dye (Bio-Rad) to determine protein concentration in the lysates, and 500 μg of protein lysate diluted in lysis buffer was loaded into streptavidin agarose beads (Thermo Scientific) and rotated overnight at 4 °C. The beads were then washed 4–6 times the next day with lysis buffer containing same reagents as above except 0.1% NP-40. The proteins were eluted by adding 50 μL of 2× Laemmli sample buffer (Bio-Rad) and boiling the samples at 95 °C for 5 min. Whole-cell lysate samples of 50 μg were eluted in 20 μL of the same Laemmli buffer and boiled for 5 min. Samples were loaded into 8% SDS-PAGE gels and run for 20 min at 80 V, and the voltage was then increased to 160 V for 1 h. Proteins were transferred from gels to 0.2 μm PVDF blotting membrane (GE Healthcare Life Sciences, 2.5 h, 0.3 mA at 4 °C). Membranes were incubated with 5% fat-free milk for 1 h at room temperature to block nonspecific background then treated with primary antibodies overnight at 4 °C as follows: rabbit polyclonal anti-A1R (1:1000, Sigma) or anti-A2AR (1:1000, Millipore). Membranes were then probed with the corresponding HRP-conjugated secondary antibody and then ECL was performed (Bio-Rad Labs). Whole-cell lysate membranes were reprobed with anti-GAPDH (mouse monoclonal, Millipore 1:1000) on the same blot to ensure consistency of loading. Analysis was performed using Quantity One Basic (Bio-Rad) and ImageJ (NIH, public domain) and data were expressed as the percentage of the intensity of biotinylated target protein (surface expression) to that of corresponding whole cell lysate [11, 12, 16].

### Electrophysiological Studies

For electrophysiology experiments, hippocampal slices were placed in an electrophysiology recording chamber and were constantly perfused with oxygenated aCSF at a rate of 3 mL/min. Field EPSPs (fEPSPs) were evoked using a bipolar tungsten stimulating electrode for orthodromic stimulation of the hippocampal Schaffer collateral pathway. A recording electrode made of a glass micropipette filled with aCSF (resistance 1–3 MΩ) was used to measure CA1 fEPSPs in *stratum radiatum*. The fEPSP signals were amplified 1000 times with an AC amplifier, bandpass filtered at 1000 Hz, digitized at 10 kHz using a Digidata 1440A digitizer (Molecular Devices, Foster City, CA), and saved using the Clampex 9.0 software (Molecular Devices). The fEPSP slopes were analyzed using Clampfit 9.0 (Molecular Devices).

fEPSPs were evoked with each sweep being 0.1 ms in length every 30 s (0.03 Hz). A stable baseline synaptic response was established for at least 20 min to ensure a stable baseline. In hypoxia experiments, oxygenated aCSF was replaced with aCSF that had been bubbled with 95% N_2_/5% CO_2_ for at least 15 min prior to the experiment to deplete the majority of oxygen in solution. All drug treatments were added directly to the aCSF solution. All recordings were normalized to the average of the final 10 sweeps (5 min) of the baseline recording period (100%).

### Propidium Iodide Staining and Fluorescence Imaging

To examine cell death in the rat hippocampus following hypoxic insult, propidium iodide (PI) was used as a fluorescent marker due to the fact that it selectively labels dead cells with a disrupted plasma membrane [12, 39]. Hippocampal slices (400 μm thickness) were incubated in appropriate drug treatments at the given time in the experiment. Slices were then transferred to hypoxic aCSF for a period of 20 min and then returned to oxygenated aCSF for a 3-h period. In the final hour, PI (5 μg/mL) was added to the aCSF solution. Following the incubation period, slices were rinsed thoroughly in aCSF (3 × 10 min) and fixed in 4% paraformaldehyde at 4 °C overnight. On the following day, slices were washed in 1× PBS (3 × 10 min) and then mounted on glass microscope slides and treated with ProLong Gold Antifade Reagent (Invitrogen/ThermoFisher Scientific, Carlsbad CA, USA) and sealed under glass microscope coverslips. Following the addition of PI to aCSF, all subsequent procedures were performed in the dark to prevent photobleaching.

Imaging was performed with a Zeiss LSM700 laser scanning confocal microscope (Carl Zeiss, Germany) using green light (543 nm) to induce PI fluorescence. The whole hippocampus was imaged in pieces using a 10× objective lens, and images of the CA1 pyramidal neuron layer were obtained using the Zeiss Plan-Apochromat 63×/1.4 oil objective lens (Carl Zeiss). 10× images were assembled into montages of the full hippocampal slice using Adobe Photoshop (Adobe Systems, San Jose CA, USA). CA1 images were acquired as Z-stack images of about 200 μm depth into the hippocampal slice with each Z-stack image taken every 1 μm along the Z-axis. Two Z-stack images were taken along CA1 for each hippocampal slice and were analyzed using densitometry analysis using ImageJ (public domain). Confocal imaging of PI stained hippocampal slices was single blinded.

### Drug Treatments

Bath applied drug in electrophysiology studies are described as follows: SCH442416 (Sigma), a selective adenosine A2A receptor antagonist; 8-cyclopentyl-1,3-dipropylxanthine (DPCPX) (Sigma), a selective adenosine A1R antagonist; dichlororibofuranosylbenzimidazole (DRB), dimethylaminotetrabromobenzimidazole (DMAT), and tetrabromobenzotriazole (TBB) (all from Tocris, Bristol, UK), selective CK2 inhibitors; spermine tetrahydrochloride (Tocris), a CK2 activator; 6-cyano-7-nitroquinoxaline-2,3-dione (CNQX) (Tocris), a potent AMPAR antagonist; 6,7-dinitroquinoxaline-2,3-dione (DNQX) (Tocris), a competitive antagonist at AMPA and kainate receptors; 5′-(2-cyanophenyl)-1′-phenyl-2,3′-bipyridinyl-6′ (1H)-one (Perampanel, E2007) (Biorbyt, Cambridge, UK), a non-competitive AMPAR antagonist; 2,3-dioxo-6-nitro-1,2,3,4-tetrahydrobenzo[f]quinoxaline-7-sulfonamide (NBQX) (Tocris), a potent AMPAR antagonist; (5R,10S)-(-)-5-methyl-10,11-dihydro-5H-dibenzo[a,d]cylcohepten-5,10-imine maleate (MK-801) (Tocris), a non-competitive NMDAR antagonist; D-(-)-2-amino-5-phosphonopentanoic acid (D-AP5 or D-APV) (Tocris), a competitive NMDAR antagonist;(S)-N-[7-[(4-aminobutyl)amino]heptyl]-4-hydroxy-α-[(1-oxobutyl)amino]benzenepropanamide dihydrochloride (Philanthotoxin-74) (Tocris), GluA1- and GluA3-containing AMPAR antagonist; and N,N,H,-trimethyl-5-[(tricyclo[3.3.1.13,7]dec-1-ylmethyl)amino]-1-pentanaminiumbromide hydrobromide (IEM 1460) (Tocris), an open channel blocker of non-GluA2-containing AMPARs. All bath applied drugs were dissolved in DMSO before adding to aCSF. In each treatment, the final concentration of DMSO is less than 0.1%. Dynasore hydrate, a GTPase dynamin inhibitor, was purchased from Sigma and also dissolved in DMSO before added to bath treatment. We showed previously that GluA2-endocytosis can be inhibited by bath applied active Tat-GluA2–3Y peptide (YG) [11]. YG peptide consists of the following amino acid sequence: YGRKKRRQRRR-869YKEGYNVYG877, where Tat is YGRK KRRQRRR (the cell-penetrating amino acid peptide sequence contained within the protein transduction domain of HIV gene called Tat), and 869YKEGYNVYG877 represents a GluA2 C-terminal amino acid sequence that interacts with the endocytic protein AP2, thus preventing GluA2 internalization [11, 40]. The YG peptide and its scrambled version (Scrambled YG: YGRKKRRQRRR-VYKYGGYNE) were purchased from DgPeptides Co., Ltd. (Hangzhou City, Zhejiang Province, China).

### Statistical Analysis

For electrophysiological recordings, the order of recording control vs. treated slices (with antagonists) were randomly chosen so that the length of time of slice preincubation was not a confounding variable. For propidium iodide staining and analysis, the hippocampal slices were independently stained and confocal imaging and analysis were independently performed by another individual. Minimum group sizes (one slice per animal) were estimated based on power calculations using previously obtained means and variances from similar recording conditions from our lab. We also performed post hoc power analysis using the GraphPad Prism software to confirm that our group sizes (*n* values) provided the appropriate number for good statistical power.

Results are expressed as mean ± SEM. Graphing and statistical analysis were performed using the GraphPad 6.0 software (GraphPad). Densitometry of PI staining was performed using ImageJ (public domain). Statistical significance was assessed using one-way ANOVA with the Tukey–Kramer post hoc test with 95% confidence interval using the GraphPad Prism 6 software (GraphPad, La Jolla, CA, USA). Student’s paired *t* test was also used when comparing two treatment groups. Numbers of experiments are indicated by *N*. The reported *N* values in figure legends of fEPSP recordings, Western blotting, and PI staining were obtained from independent experiments in which hippocampal slices were obtained from brains of different animals and randomly used for each recording. Probability values (*P*) of less than 0.05 were considered statistically significant.

## Results

### A1R Antagonist Inhibited the Hypoxia/Reperfusion fEPSP Biphasic Responses, Whereas A2AR Inhibition Prevented Only the APSP

Since prolonged A1R activation led to decreased A1R surface expression but increased A2AR surface expression, increasing the excitatory effect of A2ARs [11], therefore, we hypothesized that A1R inhibition would prevent not only the hypoxia-induced synaptic depression but also the expression of APSP. Moreover, we predicted that A1R antagonism would attenuate both the hypoxia-induced reduction of A1R and the increase in A2AR surface expression, resulting in subsequent inhibition of APSP. Using acute hippocampal slices, fEPSP recordings were performed using a 20-min hypoxic insult followed by a 45-min normoxic washout period. Slices were pretreated with either the A1R-selective antagonist DPCPX (100 nM) [41] or the A2AR-selective antagonist SCH442416 (5 nM) [42]. Treatment of hippocampal slices with DPCPX significantly attenuated hypoxia-induced synaptic depression and fEPSPs showed comparable levels to baseline before inducing hypoxia; however, synaptic transmission was ≈ 80% attenuated during hypoxia with slices treated with either control (DMSO) or the A2A receptor antagonist SCH442416. This observation confirms the crucial role of elevated extracellular adenosine in mediating a short-term neuroprotective effect following ischemia through A1R-inhibition of neuronal excitability and presynaptic glutamate release [4, 5]. In contrast, normoxic reperfusion of hippocampal slices following the 20-min hypoxia showed marked increase in synaptic transmission (150% of baseline) that was prevented by either A1R or A2AR antagonism. Therefore, the biphasic response of hypoxia/reperfusion consists of two phases: it starts with A1R-dependent synaptic depression during hypoxia followed by A2AR-dependent potentiation of fEPSP during normoxic reperfusion, that we have termed adenosine-induced post-hypoxia synaptic potentiation (APSP). This also suggests a functional link between A1Rs and A2ARs, whereby a prior prolonged A1R activation is required for A2AR upregulation, inducing APSP.

### CK2 Inhibition Differentially Regulated A1R and A2AR Surface Expression in Normoxic Condition But Downregulated Both A1R and A2AR in Hypoxia in Rat Hippocampus

Previous studies showed that casein kinase 2 (CK2) oppositely modulates the G protein-coupled D1 and D2 receptors [22–24, 43]. CK2 negatively regulates the Gα_S_-coupled D1 receptor, while CK2 activity upregulates the Gα_i_-coupled D2 receptors [22–24, 43]. Moreover, CK2 is known to play an important role in negative regulation of the excitatory A2ARs, another Gα_S_-coupled G protein-coupled receptor (GPCR) [22]. Thus, we hypothesized that CK2 might be involved in the reciprocal regulation of the GPCRs A1Rs and A2ARs. To test our hypothesis, we firstly treated hippocampal slices in regular normoxic condition with either the CK2 inhibitor DRB (100 μM) or the CK2 activator spermine (300 μM). The concentration of DRB used in the current study (100 μM) has been shown to inhibit CK2 activities in rat acute hippocampal slices [44]. Then, we performed surface biotinylation assays and Western blotting to quantify the surface levels of A1Rs and A2ARs. Our results (Fig. 2) confirmed that CK2 inhibition with DRB showed 50% increase in surface expression of the Gα_S_-coupled A2AR, whereas the CK2 activator spermine resulted in downregulation of A2ARs compared to the control hippocampal slices treated with DMSO. These results are consistent with previous findings by Rebholz and colleagues that suggest CK2 negatively regulates Gα_s_-coupled GPCRs [22]. In marked contrast, hippocampal slices treated with the CK2 inhibitor DRB showed significant decrease in surface levels of the inhibitory Gα_i_-coupled A1R, while CK2 activation with spermine caused marked elevation of A1R surface expression (Fig. 2a). Since spermine and other polyamines are known to increase the function of NMDA receptors by binding to an extracellular region of the receptor [45], we incubated hippocampal slices with the NMDA receptor antagonist D-APV for 30 min before applying spermine in order to rule out the possible confounding effect of NMDA receptor-mediated upregulation by spermine. Interestingly, pretreatments of hippocampal slices with D-APV did not prevent the effects of spermine-induced changes in the surface levels of A2AR or A1R. Consistent with the previous findings by Rebholz and colleagues [22–24], our results suggest that CK2 activators may facilitate A2AR desensitization (i.e., increased receptor endocytosis) but reduce A1R internalization (i.e., reduced receptor endocytosis) in normoxic conditions.

**Fig. 1:**
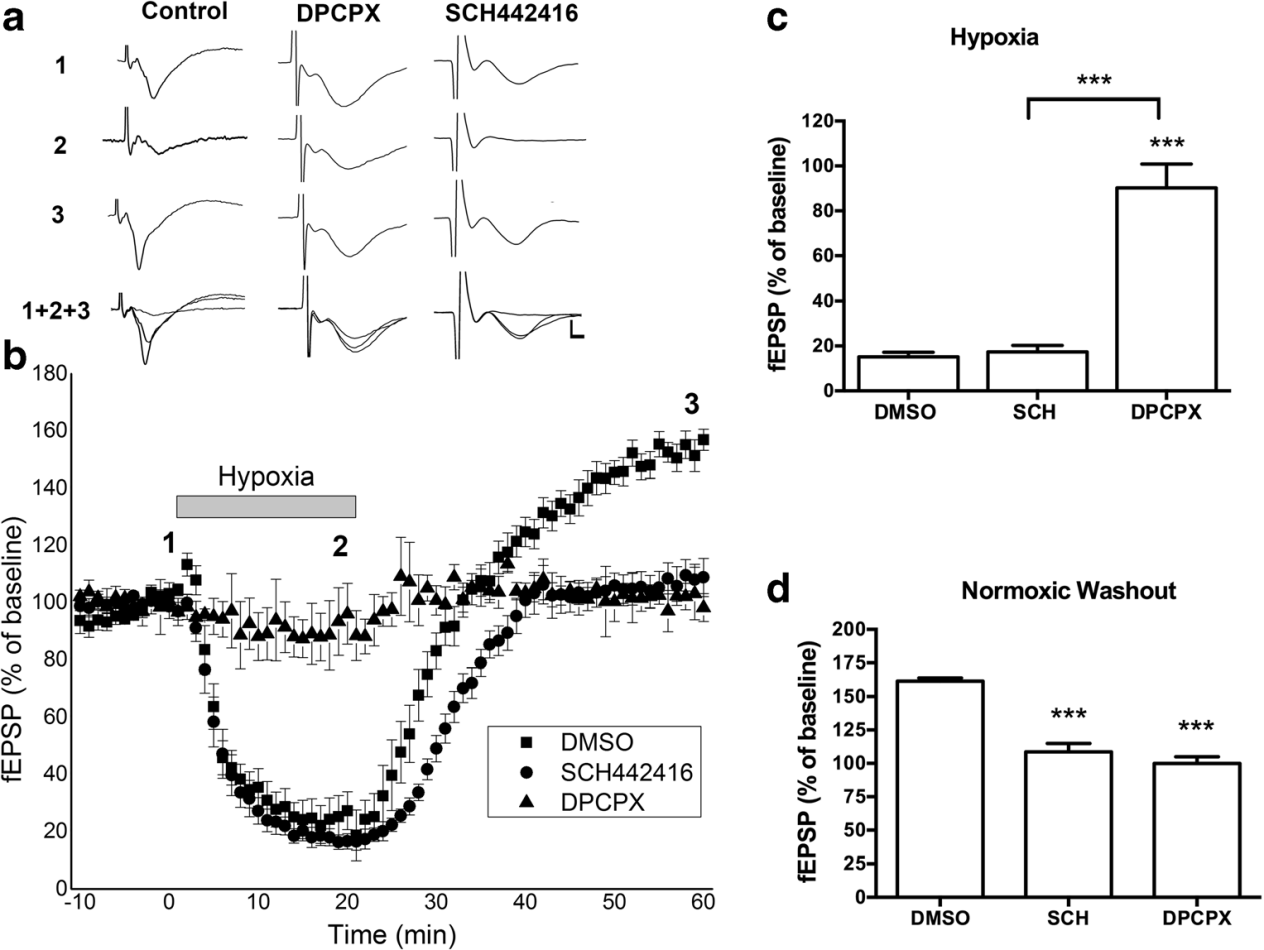
Inhibition of A2ARs prevented adenosine-induced post-hypoxia synaptic potentiation (APSP), whereas inhibition of A1Rs prevented both hypoxia-induced synaptic depression and APSP. Hippocampal slices were treated with the A2AR antagonist SCH442416 (SCH, 5 nM) or the A1R antagonist DPCPX (100 nM) 30 min prior to the start of the experiment and were continuously perfused with the appropriate treatment throughout the course of the experiment. Hypoxia/reperfusion induced a biphasic response in fEPSP, starting with hypoxia-induced synaptic depression and then followed by 50% enhancement of synaptic transmission we referred to as APSP. The A1R antagonist DPCPX pretreatment prevented the biphasic response of hypoxia/reperfusion, whereas the A2AR antagonist SCH442416 only prevented the development of APSP. **a.** Representative hippocampal fEPSP traces for each treatment showing sample traces of the last 10 sweeps (final 5 min) of the baseline recording (1), the end of the 20-min hypoxia period (2), and then end of the 40-min normoxic washout period (3), and an overlay of the three traces together (1 + 2 + 3). Scale bars show 10 ms (*x*), 0.5 mV (*y*). **b.** Time course plots of the average fEPSP slopes which were normalized to the baseline value (100%) of each recording. **c.** Summary bar graph showing the mean normalized fEPSP slope percentage of the final 5 min (10 sweeps) of the 20-min hypoxia period. Both the control (DMSO) and SCH-treated slices showed over 80% synaptic depression, whereas DPCPX-treated slices showed almost no synaptic depression due to hypoxia. **d.** Summary bar graph showing the mean normalized fEPSP slopes of the final 5 min (10 sweeps) of the normoxic washout period. Control (DMSO) slices showed about 165% synaptic potentiation over this period (APSP) compared to baseline, which was prevented by both A1R and A2AR inhibition (DPCPX and SCH, respectively), which only recovered to baseline levels (100%) without any APSP. *N* = 7 for control slices, *N* = 5 for DPCPX slices, and *N* = 7 for SCH442416 slices from different rats. Graphed values show mean ± SEM. Significance values: ***p < 0.001

**Fig. 2:**
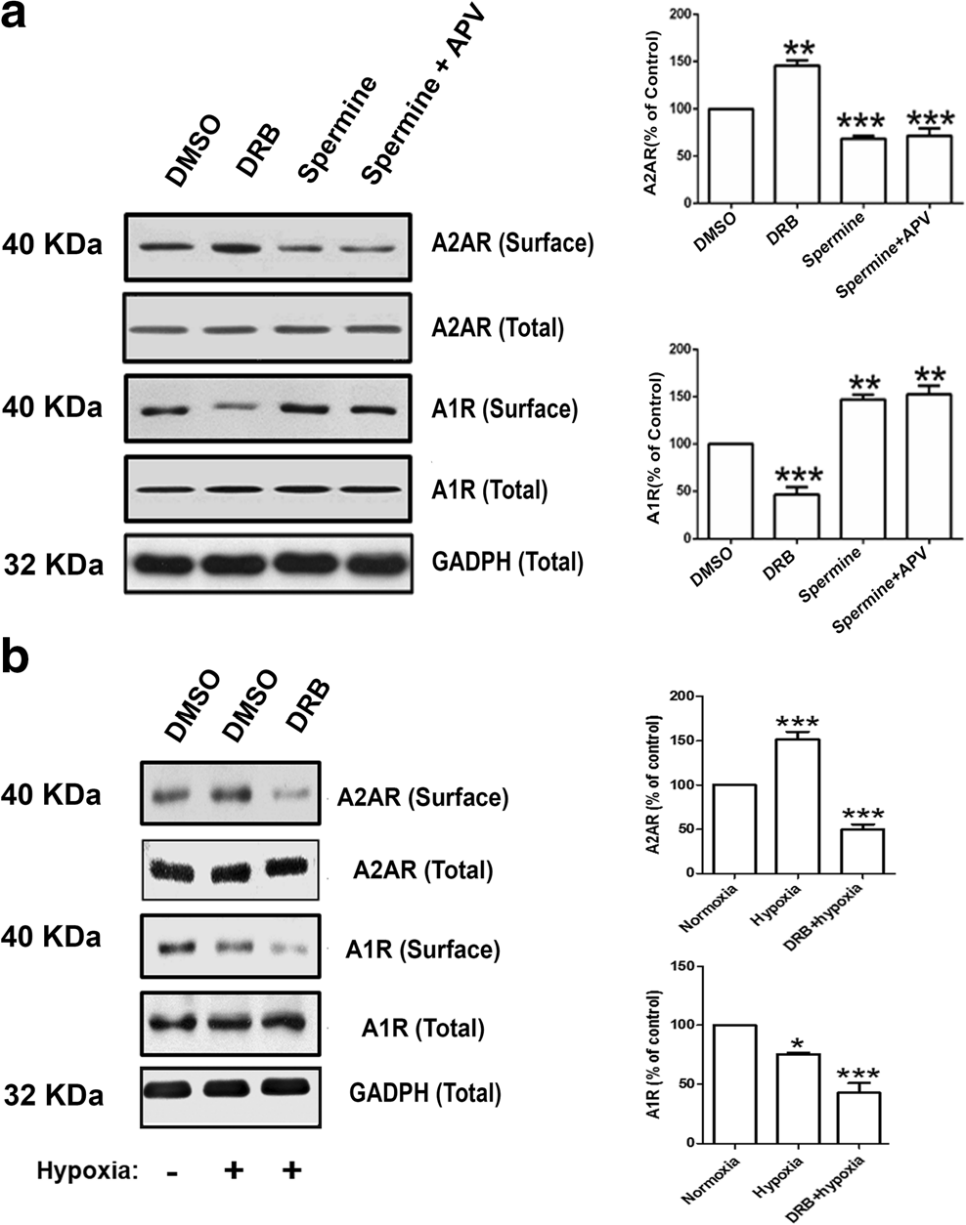
CK2 inhibition oppositely regulated A1R and A2AR in normoxic condition but caused profound downregulation of both A1R and A2AR in hypoxia in rat hippocampus. **a.** Hippocampal brain slices were pretreated with DMSO (control), or CK2 inhibitor DRB (100 μM), or CK2 agonist spermine (300 μM) either alone or with D-APV (50 μM) for 30 min before adding spermine. After 1 h treatment, hippocampal slices were biotinylated (see the “Methods” section for details). The CK2 inhibitor DRB enhanced A2AR surface levels and decreased A1R surface expression, while the CK2 activator spermine decreased A2AR and increased A1R surface levels. Representative blots of surface proteins (biotinylated) and total cell lysates probed for A1R and A2AR antibody are shown (left panel) with the corresponding densitometry values (right panel). The whole lysate blots did not show significant changes in densitometry values in all treatment groups after normalization to GAPDH and were used to normalize all surface (biotinylated) values. Normalized values were then expressed as percentage of normoxia values (taken as 100%). **b.** After 20-min hypoxia, brain slices were immediately subjected to biotinylation to assess the relative abundance of A1R and A2AR. The CK2 inhibitor DRB decreased surface levels of both A1R and A2AR after 20 min hypoxia, while hypoxia alone downregulated A1R but upregulated A2AR. Representative blots of surface and total cell lysates probed for A1R and A2AR antibody were quantified by densitometry as in **a**. Values in summary bar charts are means ± SEM from *N* = 4 independent experiments (*p < 0.05, **p < 0.01, and ***p < 0.001)

Since A1R and A2AR regulate APSP levels during normoxia/reperfusion (Fig. 1) and both receptors are oppositely regulated by CK2 (Fig. 2a), we hypothesized that CK2 inhibition may alter receptor endocytosis during hypoxia. As shown in Fig. 2b, hypoxia alone significantly decreased surface levels of the inhibitory A1R by ~ 20% compared to normoxia, which is consistent with previous studies showing significant A1R desensitization after hypoxia [38, 46–48]. However, in the presence of the CK2 inhibitor DRB, the hypoxia-induced A1R downregulation was even more pronounced. In contrast to A1R, hypoxia markedly increased the surface levels of A2AR by ~ 50% compared to control normoxia (Fig. 2b). In the presence of DRB, however, hypoxia significantly decreased A2AR surface levels approximately 50% below normoxia levels. The levels of adenosine receptors remained constant in whole hippocampal lysates in all treatments (bottom panels of Fig. 2a, b), suggesting that protein degradation is unlikely to contribute to these dynamic changes in adenosine receptor surface expression during a 20-min hypoxic insult with or without CK2 inhibition. Collectively, our results indicate that A1Rs and A2ARs are functionally linked by extracellular adenosine levels and by intracellular CK2 activities.

### CK2 Inhibition Attenuated Both Hypoxia-Induced fEPSP Biphasic Responses and Hippocampal Cell Death in the CA1 Region of Rat Hippocampus

**Fig. 3:**
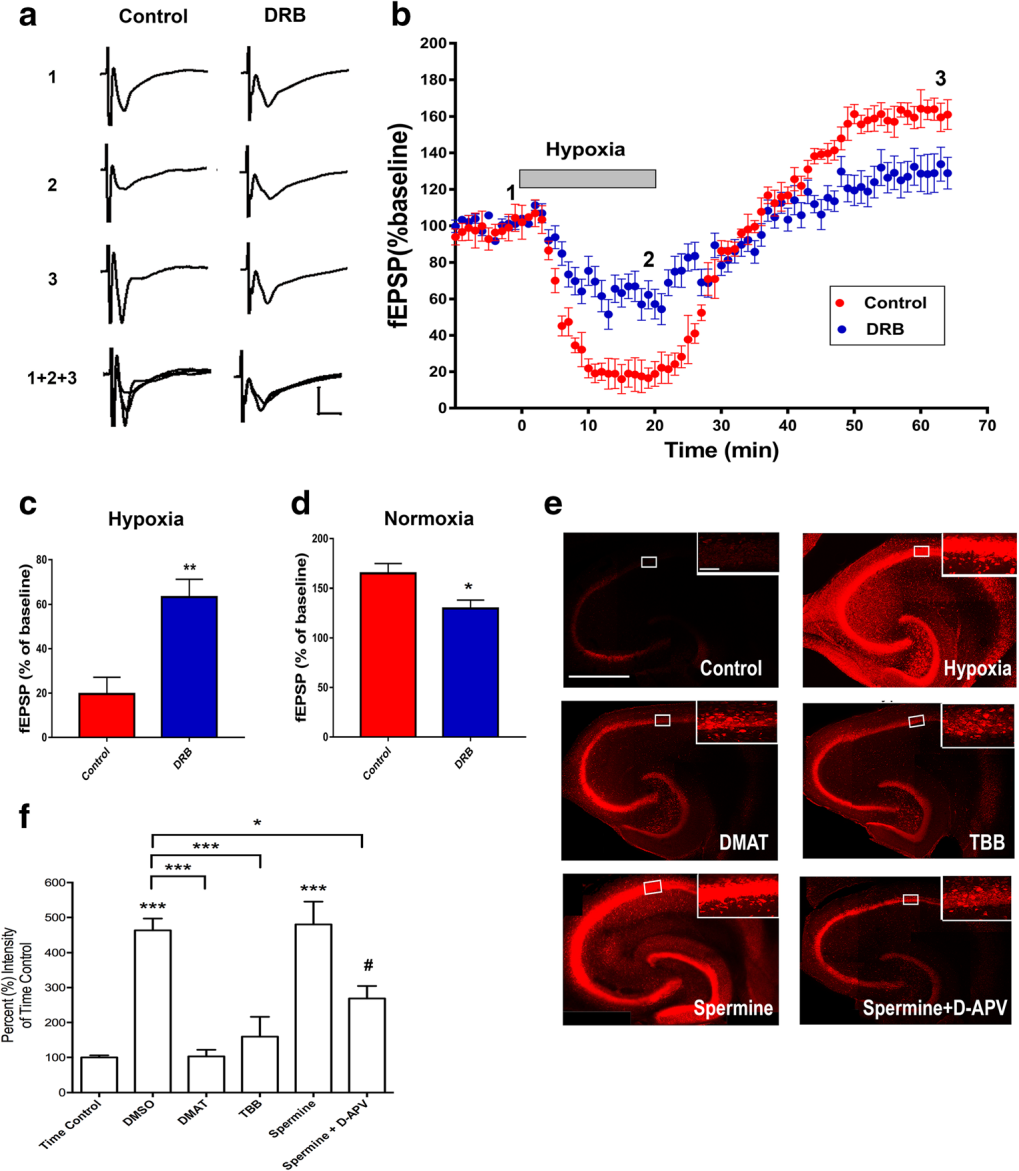
CK2 inhibition attenuated both hypoxia/reperfusion-induced APSP and hypoxia-induced hippocampal cell death in the CA1 region of rat hippocampus. Hippocampal slices were treated with either DMSO (control, *N* = 12) or DRB (CK2 inhibitor, 100 μM, *N* = 8) 1 h prior to the start of the experiment and were continuously perfused with the appropriate treatment throughout the course of the experiment. **a.** Representative hippocampal fEPSP traces for each treatment showing sample traces of the last 10 sweeps (final 5 min) of the baseline recording (1), the end of the 20-min hypoxia period (2), and the end of the 40-min normoxic washout period (3) and an overlay of the three traces together (1+2+3). Scale bars show 10 ms (*x*), 0.5 mV (*y*). **b.** Time course plots of the average fEPSP slopes which were normalized to the baseline value (100%) of each recording. **c.** Summary bar graph showing the mean normalized fEPSP slope percentage of the final 5 min (10 sweeps) of the 20-min hypoxia period. DMSO-treated slices showed over 80% synaptic depression, whereas DRB-treated slices showed 35% synaptic depression. **d.** Summary bar graph showing the mean normalized fEPSP slopes of the final 5 min (10 sweeps) of the normoxic washout period. Control (DMSO) slices showed about 165% synaptic transmission above baseline over this period (APSP), compared to only 135% synaptic transmission above baseline in the presence of DRB. **e.** Representative images taken at 10× magnification of full hippocampal slices subjected to hypoxia and treated with DMSO, DMAT (5 μM), TBB (10 μM), spermine (300 μM), and spermine (300 μM) + D-APV (50 μM), respectively. Smaller panel in the top right corner of each large panel shows an image of the CA1 cell layer (in white boxed region of large panel) taken at 63× magnification. The small panels of area CA1 were analyzed to compare relative PI intensity and normalized to the time-matched controls (100%). Scale bars: 1 mm (whole hippocampus, large panel) and 10 μm (CA1, in the top right corner). **f.** Summary bar graph showing values of average intensity normalized to time control values (100%), *N* = 5 independent experiments, 5 rats per treatment. Hippocampal slices treated with DMSO or the CK2 activator spermine showed significant hippocampal cell death following hypoxia/reperfusion. In contrast, inhibition of CK2 with DMAT or TBB markedly inhibited hypoxia-induced cell death, i.e., the propidium iodide staining under CK2 inhibitor-hypoxia treatment was comparable to that in time-matched control slices without hypoxia. In addition, the NMDA receptor antagonist D-APV attenuated the hypoxia-induced neuronal damage in the presence of spermine. Graphed values show mean ± SEM. Significance values: *p < 0.05, **p < 0.01, and ***p < 0.001 compared to control; ^#^*p* < 0.05 compared to spermine

To examine the functional effects of the observed altered levels of surface expressed adenosine A1Rs and A2ARs following a 20-min hypoxic insult and to determine whether pretreatment of hippocampal slices with the CK2 inhibitor DRB has any impact on APSP levels, we performed fEPSP electrophysiology recordings. As shown in Fig. 3, DRB (100 μM) pretreatment for 1 h before subjecting hippocampal slices to a 20-min hypoxic insult (DRB, blue filled circles) significantly reduced hypoxia-induced synaptic depression compared to control DMSO (red filled circles). This result suggests that CK2 inhibition, which reduced A1R surface expression both before and during hypoxic stimulation (Fig. 2), can attenuate A1R-induced synaptic depression during hypoxia. Moreover, CK2 inhibition attenuated the APSP levels during the normoxic reperfusion (i.e., only produced 25% APSPs above baseline compared to 50–60% without CK2 inhibitor DRB; Fig. 3d). Together, these results suggest that (1) CK2 inhibition attenuated hypoxia-induced synaptic depression likely owing to the CK2 inhibitor-mediated reduction in A1R surface expression and (2) CK2 inhibition reduced APSP levels consistent with the observed reduction in A2AR surface expression after 20 min hypoxia.

The role of CK2 as a potential neuroprotective protein has been explored [49], but the direct impact of CK2 inhibition or activation on neuroprotection or neurodegeneration in hypoxic injury has not been well established. Since CK2 inhibition attenuated both synaptic depression during hypoxia and APSP during normoxic/reperfusion following the hypoxia, we then determined whether CK2 inhibition could prevent hippocampal cell death mediated by hypoxia. To test the effect of CK2 inhibition in hypoxia-induced cell death, hippocampal slices were pretreated with DMAT or TBB, which are both potent CK2 inhibitors, or with the CK2 activator, spermine alone, or combined with D-APV to prevent spermine-induced activation of NMDA receptors as previously mentioned. Following a 20-min hypoxic insult, slices were reintroduced to normoxic conditions and incubated for 3 h prior to fixation, during which propidium iodide (PI) was added to label dead cells in the last hour as described in the “Methods” section. As shown in Fig. 3e, f, inhibition of CK2 with either DMAT or TBB showed significantly less hippocampal cell death following hypoxia/normoxic reperfusion compared to hippocampal slices treated with vehicle control DMSO. While inhibition of CK2 with DMAT or TBB treatment prior to hypoxia was shown to attenuate the hypoxia-induced cell death, pharmacological activation of CK2 with spermine, however, did not cause a further potentiation of hypoxia-induced hippocampal cell death. Instead, spermine displayed similar levels of cell death after hypoxia as DMSO-treated hippocampal slices (Fig. 3e, f). Co-administration of the NMDAR antagonist D-APV with spermine significantly attenuated the hypoxia-mediated hippocampal cell death, indicating that hypoxia-induced hippocampal cell death in the absence or presence of spermine was in part caused by increased NMDAR activity. These data suggest that inhibition of CK2 activity may provide neuroprotection to hippocampal neurons during hypoxic conditions.

### APSP Is Dependent on Hypoxia-Induced, Clathrin-Mediated GluA2-Internalization

**Fig. 4:**
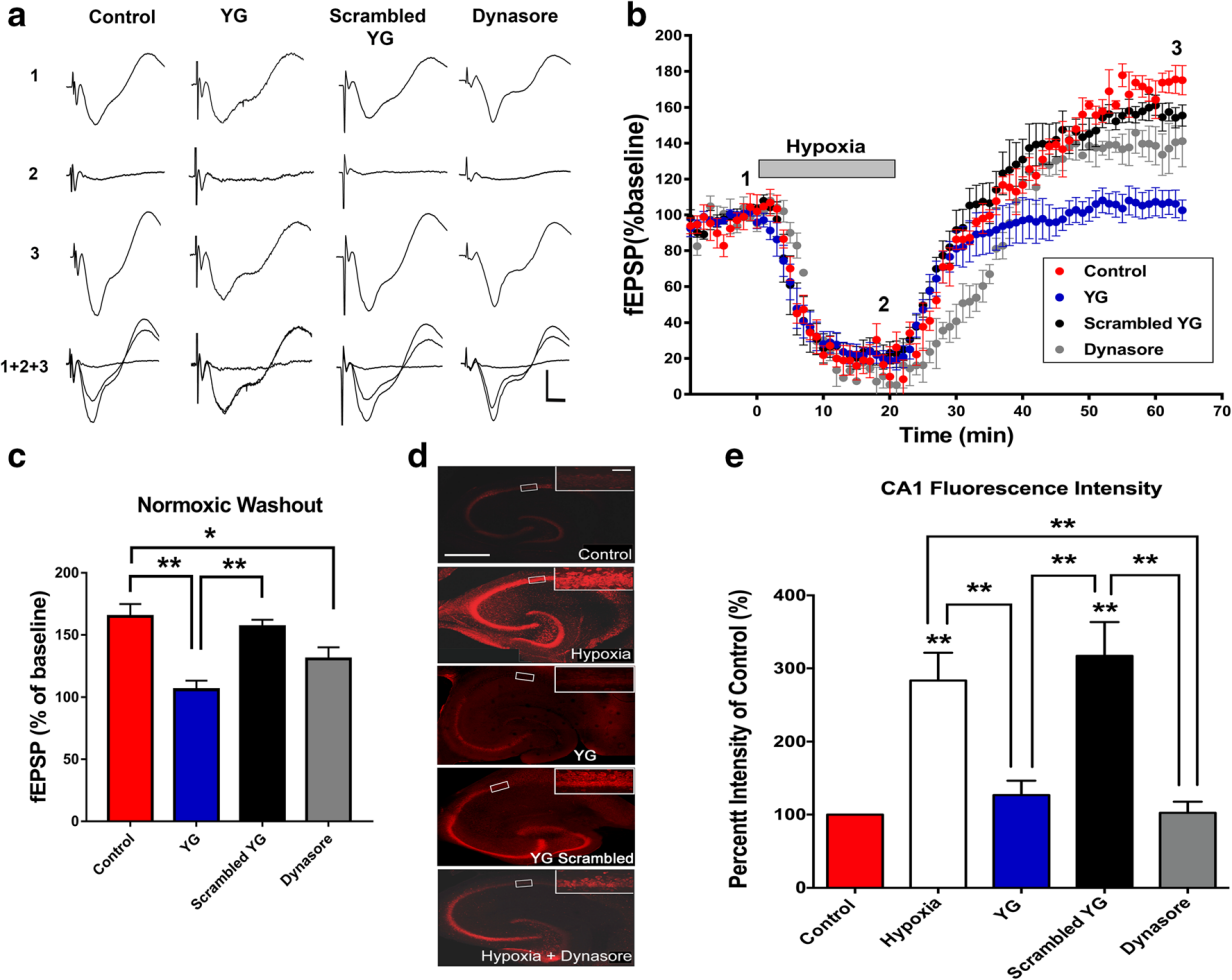
APSP and hippocampal neuronal death involve hypoxia-induced clathrin-mediated and dynamin-dependent GluA2 AMPAR internalization. Hippocampal slices were treated with DMSO (vehicle control), Tat-YG peptide inhibitor (YG, 2 μM), scrambled YG (2 μM), or dynasore (50 μM) for 1 h prior to the start of experiments and subsequently were perfused with these agents throughout the course of the experiment. **a.** Sample fEPSP traces from control (DMSO), YG, scrambled YG, and dynasore-treated hippocampal slices showing the average of the last 5 min (10 sweeps) of the baseline recording (1), 20-min hypoxia (2), 45-min normoxic washout (3), and an overlay of the three (1+2+3). Scale bars show 10 ms (*x*), 0.5 mV (*y*). **b.** Time course plot showing the mean fEPSP slope values as a percentage of the baseline (normalized to 100%) throughout the course of the experiment. **c.** Bar graph showing the mean fEPSP values during the final 5 min (10 sweeps) of the 45-min normoxic washout period. Compared to control, dynasore-treated slices showed significantly less APSP in the normoxic washout period while YG completely prevented the development of APSP during normoxic washout. **d.** Representative fluorescent confocal microscopy images of hippocampal slices treated with hypoxia (as in Fig. 3e). Large panels show representative images of the full hippocampus for each treatment group. Smaller panel in the top right corner of each large panel shows an image of the CA1 cell layer (in white boxed region of large panel) taken at 63× magnification. The small panels of area CA1 were analyzed to compare relative propidium iodide (PI) intensity and normalized to the time control (100%). Scale bars: 1 mm (whole hippocampus, large panel) and 10 μm (CA1, in the top right corner). **e.** Bar graph showing mean fluorescence intensity values of hippocampal slices stained with PI. Fluorescence intensity was measured using confocal fluorescent images taken in area CA1 of hippocampal slices at 63× magnification. Hypoxia caused a 200% increase in PI intensity compared to the time-matched control (no hypoxia) slices, indicating increased cell death caused by hypoxia. Pretreatment with dynasore or Tat-YG peptide inhibitor prior to hypoxia produced significant inhibition of the hypoxia-induced cell death to levels comparable to control slices. In contrast, the scrambled Tat-YG inactive peptide inhibitor failed to attenuate hypoxia-induced hippocampal cell death. *N* = 6 independent experiments for each fEPSP treatment group, *N* = 6 independent experiments for PI stained slices. All graphs show either mean fEPSP percentage or PI intensity ± SEM. Significance: *p < 0.05, **p < 0.01

To examine the role of clathrin-mediated, dynamin-dependent endocytosis pathway in hypoxia-induced synaptic depression and APSP, we used the small molecule dynamin inhibitor dynasore [50]. Furthermore, we used YG peptide (2 μM) that has been previously used to block the clathrin-mediated endocytosis of GluA2 [11, 16, 40]. We previously showed that A1R stimulation induced clathrin-mediated AMPAR endocytosis [11]. Since treatment with DPCPX inhibited APSP, it was important to test whether the inhibitory effect of A1R antagonism on APSP was dependent on clathrin-mediated endocytosis. Following a 1-h pretreatment with either YG peptide or its scrambled version (2 μM), hippocampal slices were then perfused with 1 μM of the respective peptides during the entire recording period. Hippocampal slices were exposed to the same hypoxic insult and washout as above. The fEPSP recordings showed that YG-treated slices (Fig. 4b, blue filled circles) did not affect the hypoxia-induced synaptic depression, similar to the effects observed with untreated hippocampal slice (Fig. 4b, filled red symbols) or scrambled YG (Fig. 4b, filled black symbols). In contrast, YG-treated hippocampal slices did not develop APSP (Fig. 4b, c). Moreover, 1-h preincubation of brain slices with the dynamin inhibitor dynasore (50 μM) (Fig. 4b, gray filled symbols) was also effective in attenuating the APSPs, although this treatment only reduced APSPs by ~ 25%. Together, these results suggest that a prior clathrin-mediated endocytosis of GluA2 AMPAR subunits during hypoxia and other dynamin-independent endocytosis pathway may be involved in regulating APSP levels.

Next, we performed propidium iodide (PI) staining to label dead cells in the hippocampus using the same hypoxia experiment as described above (Fig. 3). Fluorescence images in both full hippocampal slices (10× magnification, Fig. 4d) and the CA1 cell layer (on the top right corner of Fig. 4d) treated with hypoxia alone or with scrambled YG peptide showed ~ 150% increase of PI fluorescence compared to control slices not subjected to hypoxic insult. On the other hand, both dynasore and YG peptide markedly attenuated hippocampal cell death induced by hypoxia/normoxia reperfusion (Fig. 4d, e), suggesting that preventing dynamin-dependent, clathrin-mediated endocytosis of GluA2 during hypoxia [11, 12] reduced hypoxia-induced hippocampal cell death. Taken together, these results suggest that APSP and hippocampal neuronal damage involve dynamin-dependent and clathrin-mediated GluA2 endocytosis.

### Synaptic Transmission in Rat Hippocampal CA1 and APSP Following Hypoxia Are Mediated Mostly by AMPARs and Not NMDARs

It has been previously reported that hippocampal synaptic transmission is mainly mediated by AMPARs with minimal contribution of NMDARs [51]. In addition, our results described above (Fig. 4) and our previous report suggest that the synaptic response to hypoxia is dependent on the A1R-mediated internalization of GluA1- and GluA2-containing AMPARs via clathrin-mediated, dynamin-dependent endocytosis [11]; thus, we hypothesized that AMPARs and NMDARs might contribute to the observed APSP following hypoxia/reperfusion. First, to test our hypothesis, we performed fEPSP experiments with multiple AMPAR and NMDAR antagonists for 30 min following a stable baseline recording. We confirmed previous findings [51] that synaptic transmission in CA1 region of hippocampus is mainly mediated by AMPAR and not by NMDAR. We found that AMPAR antagonists including CNQX, DNQX, NBQX, and perampanel (all 10 μM) abolished approximately 95% of fEPSPs after 30 min. In contrast, the NMDAR antagonists MK-801 (5 μM) and D-APV (100 μM) did not significantly affect fEPSP amplitudes after 30-min drug treatment (Supplementary Figure 1).

**Fig. 5:**
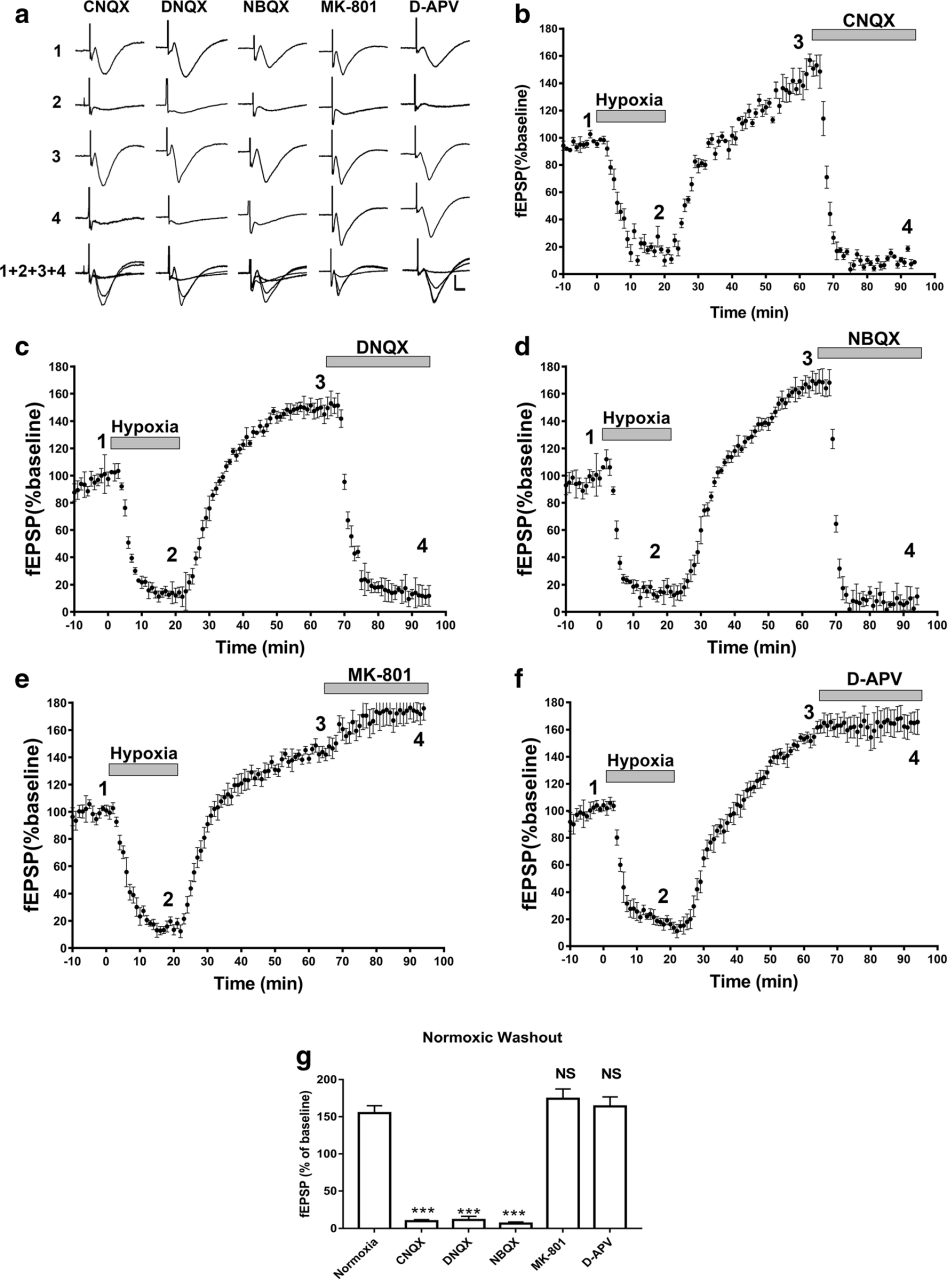
Broad-spectrum AMPAR antagonists, but not NMDAR antagonists, abolished APSPs during hypoxia/reperfusion of hippocampal slices. Hippocampal slices were treated with CNQX (10 μM), DNQX (10 μM), NBQX (10 μM), MK-801 (5 μM), or D-APV (100 μM) for 30 min following the 45-min normoxic reperfusion/washout period following hypoxia. **a.** Representative sample fEPSP traces showing the average of the last 5 min (10 sweeps) of the 10-min baseline (1), 20-min hypoxia treatment (2), 45-min normoxic washout (3), 30-min AMPAR/NMDAR antagonist treatment (4), and an overlay of all four traces (1+2+3+4). Scale bars show 10 ms (*x*), 0.5 mV (*y*). **b.**–**f.** Time course plots showing the mean fEPSP slope values as a percentage of the baseline (normalized to 100%) of hippocampal slices treated with CNQX, DNQX, NBQX, MK-801, or D-APV, respectively. **g.** Bar graph showing mean fEPSP values as a percentage of the baseline (100%, before hypoxia onset) in the last 5 min (10 sweeps) of the normoxic washout in the absence (normoxia) or presence of AMPAR or NMDAR antagonists applied for 30 min. The three AMPAR antagonists CNQX, DNQX, and NBQX all completely inhibited APSP and showed 95% inhibition of synaptic transmission; all AMPAR antagonists abolished control fEPSPs (prior to hypoxia treatment, see Supplementary Figure 1). In contrast, the NMDAR antagonist MK-801 or D-APV did not alter APSP, suggesting that APSP is mainly mediated by AMPAR. All graphs show mean ± SEM. Significance: NS, non-significant; ***p < 0.005. *N* = 7 recordings per group

Next, to test the role of AMPARs and NMDARs in modulating hypoxia-induced changes in synaptic transmission and subsequent APSP during normoxic washout, we perfused hippocampal slices with AMPAR antagonists CNQX (10 μM), DNQX (10 μM), and NBQX (10 μM) or NMDAR antagonists MK-801 (5 μM) and D-APV (100 μM) after the induction of APSP. Indeed, the AMPAR antagonists CNQX, DNQX, and NBQX all abolished fEPSPs when applied after the 45 min of normoxic/reperfusion following the hypoxic insult (Fig. 5b–d, g); however, the two NMDAR antagonists, MK-801 and D-APV, failed to block or abolish APSPs. Instead, we observed a moderate but not significant enhancement of fEPSPs with both NMDAR antagonist treatments (Fig. 5e–g). These results suggest that under our recording conditions, AMPARs mediate the hypoxia/reperfusion-induced APSP, while NMDARs play little or no role in APSP generation.

### Ca^2+^-Permeable AMPAR (CP-AMPAR) Antagonists Had No Significant Effect When Applied After APSP Induction

**Fig. 6:**
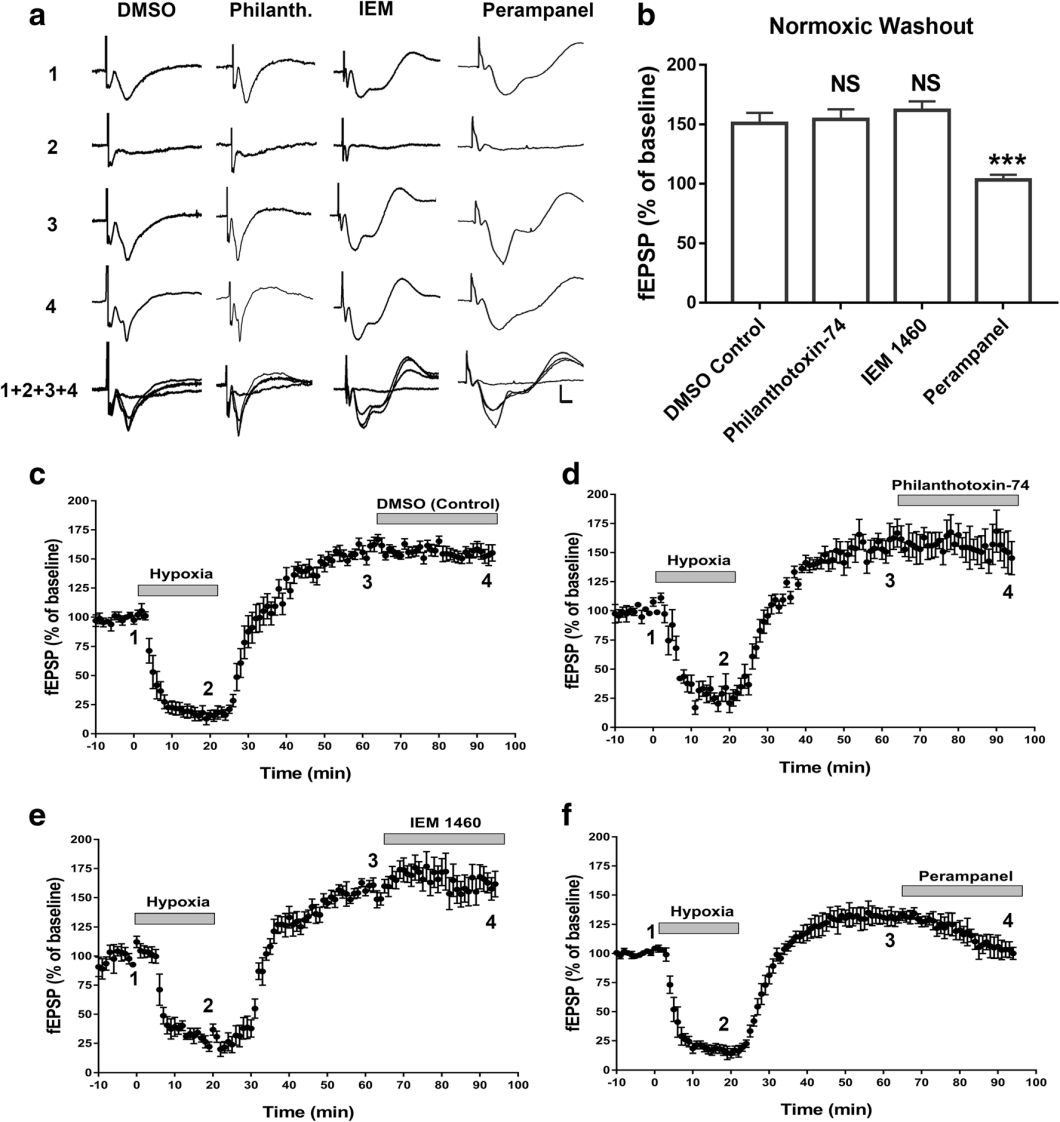
The non-competitive AMPAR antagonist perampanel, but not Ca^2+^-permeable AMPAR (CP-AMPAR) antagonists (Philanthotoxin-74 or IEM 1460), completely reversed APSPs when applied after APSP induction. Hippocampal slices were treated with Philanthotoxin-74 (50 μM), IEM 1460 (50 μM), or perampanel (200 nM) after the 45-min normoxic washout following 20-min hypoxia. **a.** Sample fEPSP traces showing the average of the last 5 min (10 sweeps) of the 10-min baseline (1), 20-min hypoxia (2), normoxic washout (3), antagonist drug treatment (4), and an overlay of the four (1+2+3+4). Scale bars represent 10 ms (*x*) and 0.5 mV (y). **b.** Summary bar graph of the average fEPSP as a percentage of the baseline (100%) of the last 5 min (10 sweeps) of the antagonist drug treatments. **c.**–**f.** Time course plots showing the mean fEPSP slope values as a percentage of the baseline values (normalized to 100%) of hippocampal slices treated with DMSO (**c**), Philanthotoxin-74 (**d**), IEM 1460 (**e**), and perampanel (**f**) following APSP induction during the washout period. The two CP-AMPAR antagonists Philanthotoxin-74 and IEM 1460 did not attenuate APSP. In contrast, the non-competitive AMPAR antagonist perampanel completely inhibited the APSP and returned fEPSPs back to baseline levels. All values show mean ± SEM. *N* = 7 independent experiments per treatment group

Since we found that APSP was mostly mediated by AMPARs (see above) and an increase in GluA1-containing AMPAR surface expression occurred following normoxic washout period [12], we then tested whether CP-AMPARs played a dominant role in APSP. Using the same protocol as above (Fig. 5), we applied for 30 min the selective CP-AMPAR antagonists, Philanthotoxin-74 (50 μM) and IEM 1460 (50 μM), following the 45-min normoxic washout period. Unlike the non-selective AMPAR antagonists used above that completely abolished APSPs, neither Philanthotoxin-74 nor IEM 1460 significantly affected APSPs (Fig. 6a, b, d, e). These results indicate that the more selective inhibitors of CP-AMPARs are ineffective in attenuating APSPs when administered late during normoxic washout.

### The Clinically Relevant AMPAR Antagonist Perampanel Attenuated APSP When Applied After 45-min Normoxic Reperfusion

Since perampanel is a non-competitive AMPAR antagonist which is already clinically approved for the treatment of drug-resistant epilepsy [34, 35], we then determined whether this clinically relevant AMPAR antagonist was effective in inhibiting APSPs and post-hypoxia hippocampal cell death. First, we tested multiple concentrations of perampanel to determine the effective concentration that would bring fEPSPs back to baseline when applied late after APSP induction without causing further synaptic depression. Unlike the CP-AMPAR inhibitors tested above that failed to inhibit APSPs when applied after the normoxic reperfusion, perampanel administration significantly attenuated the level of APSPs in a concentration-dependent manner (see Supplementary Figure 2). We found that 200 nM perampanel was sufficient to reverse APSPs back to baseline fEPSP levels (Fig. 6a, b, f). This agrees with the known IC_50_ of 230 nM perampanel for inhibition of fEPSPs at CA1 area of hippocampus [52].

### Early Inhibition of AMPARs with Either CP-AMPAR Antagonists or Perampanel Prevented Hippocampal APSPs

**Fig. 7:**
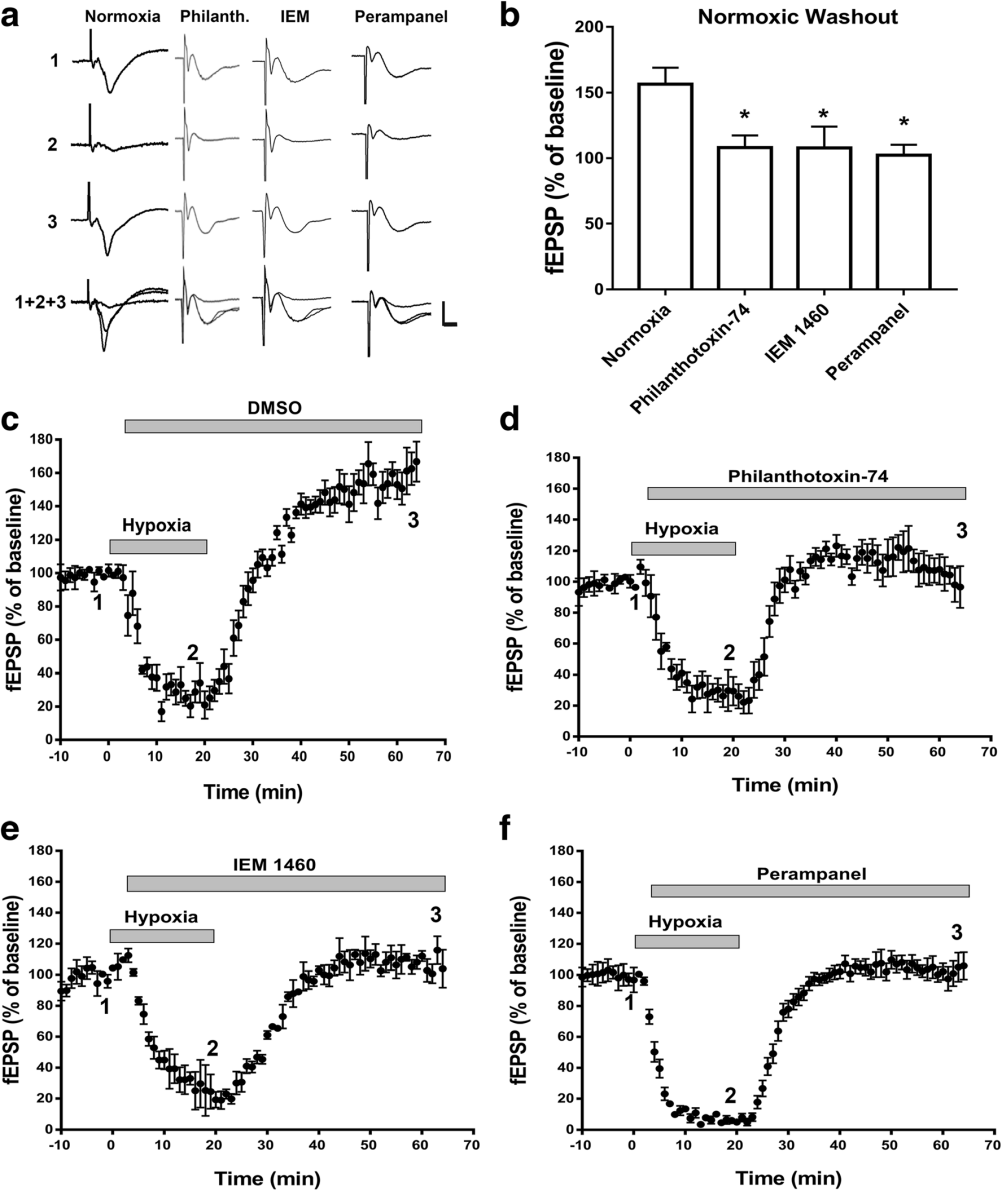
CP-AMPAR antagonists or perampanel applied at 5 min of hypoxia onset, prevented APSP induction. Hippocampal slices were treated with Philanthotoxin-74 (50 μM), IEM 1460 (50 μM), or perampanel (75 nM) 5 min after the start of the hypoxia treatment. **a.** Sample fEPSP traces showing the average of the last 5 min of the 10-min baseline (1), 20-min hypoxia (2), 45-min normoxic washout (3), and an overlay of the three (1+2+3). Scale bars show 10 ms (*x*), 0.5 mV (*y*). **b.** Summary bar graph showing the average fEPSP values as a percentage of the baseline (100%) of the last 5 min of the normoxic washout. **c.**–**f.** Time course plots showing the mean fEPSP slope values as a percentage of the baseline (normalized to 100%) of hippocampal slices treated after 5 min of hypoxia with vehicle control (**c**), Philanthotoxin-74 (**d**), IEM 1460 (**e**), and perampanel (**f**). Graphs show mean ± SEM. Significance: *p < 0.05. *N* = 7 recordings per group

Next we determined whether earlier application of CP-AMPAR antagonists would prevent the induction of APSPs. Our previous reports suggested that chronic A1R stimulation reduced A1R surface expression but increased A2AR surface expression during hypoxic/ischemic conditions, and that the levels of both GluA1- and GluA2-containing AMPAR surface expression were significantly reduced [11, 12]. In addition, upon normoxic reperfusion, GluA1 surface levels quickly recovered while GluA2 surface levels remained depressed [12], suggesting that an increased Ca^2+^ permeability of AMPARs may contribute to APSP and neuronal damage and that A2AR excitatory effects during hypoxia and subsequent normoxic reperfusion could induce rapid and transient insertion of Ca^2+^-permeable GluA1-containing AMPARs. Moreover, since we showed that stimulation of either A1R or A2AR regulates APSPs (see Fig. 1), we therefore tested the hypothesis that insertion of GluA1-containing AMPARs during the early phase of hypoxia and during normoxic reperfusion could contribute to the adenosine receptor-dependent APSP. Using Philanthotoxin-74 and IEM 1460 (same concentrations as above), we applied these inhibitors 5 min after the start of the 20-min hypoxic insult and throughout the 45-min normoxic washout. During normoxic reperfusion, both drugs prevented APSPs and returned fEPSPs back to baseline levels (Fig. 7a, b, d, e). Similar to CP-AMPAR antagonists, early treatment with a lower concentration of perampanel (75 nM vs. 200 nM) 5 min after onset of hypoxia was also effective in preventing APSPs (Fig. 7a, b, f). Together, these results suggest that delayed administrations of CP-AMPAR antagonists are ineffective in preventing APSPs and potential excitotoxicity (see below), whereas early application around the time of insult could attenuate the function of CP-AMPARs and subsequently decrease APSPs and neuronal damage. In contrast, perampanel was effective in preventing APSPs when applied early during hypoxia or late during post-hypoxia reperfusion.

### Inhibition of AMPARs with CP-AMPAR Antagonists or Perampanel 5 min After Hypoxia Onset Prevented Hypoxia-Induced Hippocampal Cell Death

**Fig. 8:**
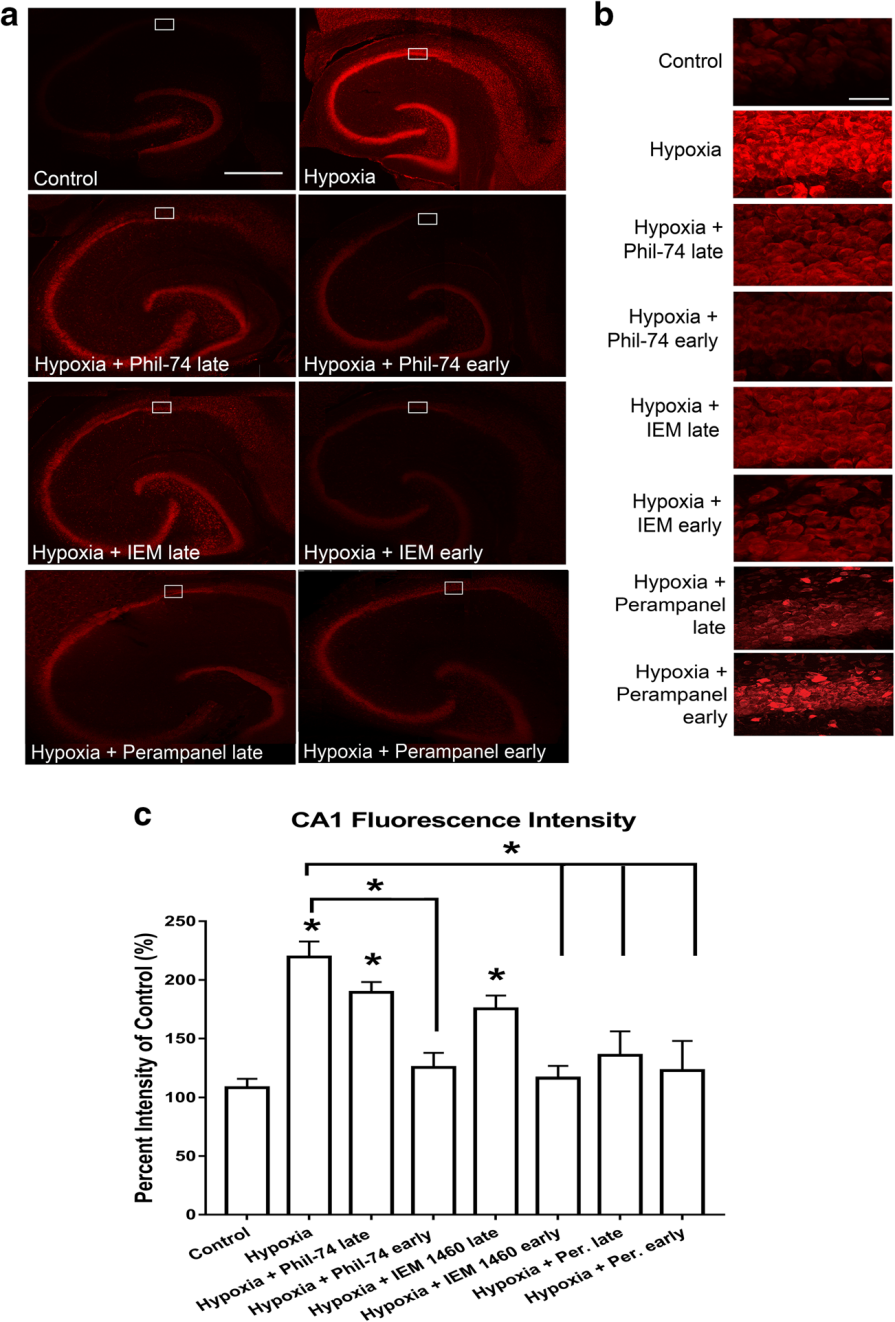
CP-AMPAR antagonists and perampanel applied 5 min into hypoxia (early) significantly attenuated hypoxia-induced hippocampal cell death. Hippocampal slices were treated with Philanthotoxin-74 (50 μM), IEM 1460 (50 μM), or perampanel (75 nM early or 200 nM late) either 5 min into hypoxia (early) or after the 45-min normoxic washout (late) and stained with propidium iodide (PI). **a.** Full hippocampal slices stained with PI taken at 10X magnification showing PI fluorescence. Increased PI fluorescence indicates increased cell death. **b.** Representative PI fluorescence images of the CA1 hippocampal cell layer (white boxed regions in **a**) taken at 63× magnification. Scale bars: 1 mm (whole hippocampus, in **a**) and 10 μm (CA1, in **b**). **c.** Bar graph showing analyzed densitometry values of the magnified CA1 images (shown in **b**) to compare relative PI fluorescence intensities between treatment groups. All AMPAR antagonists used significantly inhibited hippocampal cell death when applied 5 min after onset of hypoxia. However, only perampanel was effective in attenuating hypoxia-induced cell death when applied late following normoxic reperfusion. All values were normalized to control (100%). All values showed mean ± SEM. Significance: *p < 0.05, *N* = 6 independent experiments

To further investigate the impact of inhibiting APSPs (Figs. 6 and 7) on neuronal health, we applied CP-AMPAR antagonists (Philanthotoxin-74, IEM 1460) or perampanel at different time points and then performed PI staining to compare the levels of hippocampal damage. Early application (5 min after hypoxia) of Philanthotoxin-74 (50 μM), IEM 1460 (50 μM), or perampanel (75 nM) showed significantly less PI fluorescence compared to hypoxia alone, indicating reduced cell death (Fig. 8). However, application of CP-AMPAR antagonists after the 45-min normoxic washout period did not significantly attenuate PI fluorescence compared to hypoxia alone. In contrast, late application of perampanel (200 nM) following 45 min of normoxic reperfusion significantly attenuated the hypoxia-induced hippocampal cell death, suggesting that perampanel exhibits neuroprotection when applied early or even after delayed administration from onset of hypoxic insult.

## Discussion

In this study, we explored the functional interactions between adenosine A1 and A2A receptors and their modulation of glutamate receptor signaling and hippocampal neuronal damage in hypoxic/reperfusion injury. We showed that both A1R and A2AR are functionally linked via an intracellular protein kinase (i.e., CK2), and this adenosine receptor crosstalk mediates the induction of adenosine-induced post-hypoxia synaptic potentiation or APSP. In contrast, a previous study using a 7-min oxygen glucose deprivation (OGD) of hippocampal slices showed that OGD caused an irreversible loss of synaptic transmission that could be prevented by an A2AR antagonist [39], but these investigators never observed synaptic potentiation even after prolonged normoxic washout (> 30 min) in the presence of the A2AR antagonist ZM241385. In the present study, we observed after 20-min hypoxia treatments a significantly enhanced synaptic transmission during the 45-min normoxic washout, which was similar to the so-called “anoxia-induced long-term potentiation” that followed the 15-min hypoxia protocol used by others [19]. Our results further showed that the hypoxia-induced synaptic potentiation can be prevented by A2AR inhibition, indicating an important role of A2AR in enhancing post-hypoxia hippocampal synaptic transmission (i.e., APSPs) and neuronal damage. A previous study reported a similar potentiation of hippocampal synaptic transmission after a period of convulsions, which triggered a subsequent synaptotoxicity and A2AR-mediated convulsion-induced hippocampal neurodegeneration [53]. Interestingly, other studies have described A2AR’s control of synaptic transmission and modulation of glutamate excitotoxicity and also the therapeutic potential of A2AR antagonists for many aging-related neurodegenerative pathologies [4], including temporal lobe epilepsy [53], Parkinson’s disease [54], Alzheimer’s disease [55, 56], and stress-induced long-term potentiation (LTP) deficits [57]. It must be pointed out that the age range of the animals used in the present study (i.e., 21–30 days post weaning) may have a direct influence on APSP generation. We found in our earlier study [16] that although the total hippocampal homogenate expressions of GluA1 and GluA2 AMPARs were not significantly altered between 1- and 12-month-old hippocampal slices, the membrane and surface expressed GluA2 and GluA1 subunits were dramatically reduced (by about 50–60%) at 3, 6, and 12 months of age. Similarly, the total expression of A1R and A2AR were not significantly altered between 1 month and 12 month hippocampal slices, but the A1R surface expression was markedly reduced (by about 30%) while A2AR surface expression was upregulated (a 2-fold increase). These changes appeared to be attributed to increased levels of adenosine tone and chronic A1R stimulation in aging brains, which contributed to increased clathrin-mediated endocytosis of GluA1 and GluA2 subunits and reduced levels of LTP induction and maintenance. Therefore, these age-dependent changes in the membrane surface targeting of AMPAR subunits and adenosine receptor subtypes can, indeed, affect the levels of the post-hypoxia synaptic potentiation in older rat brains, which remains to be further investigated in future studies.

Since A1R inhibition prevented both hypoxia-induced synaptic depression and APSP induction, we determined whether the A2AR-dependent APSP required the prior A1R-mediated signaling during hypoxia-induced synaptic depression for the full induction of APSP. Collectively, our results indicate a novel functional interaction between A1R and A2AR contributing to the enhanced A2AR-mediated APSP and neuronal damage in our hypoxia/reperfusion injury model. Accordingly, we suggest that the development of hippocampal biphasic response to the hypoxia/reperfusion, namely the hypoxia-mediated synaptic depression followed by APSP, depends on a complex crosstalk between A1R and A2AR that is partially regulated by CK2. Indeed, the downregulation of A1R and subsequent upregulation of A2AR following the hypoxia/reperfusion are mimicked by administration of the CK2 inhibitor DRB. These reciprocal effects on adenosine receptors are expected to result in ablation of the A1R inhibitory effect on presynaptic glutamate release and enhancement of the synaptic transmission indirectly by the action of elevated extracellular adenosine during hypoxia/reperfusion on the upregulated A2AR, leading to increased presynaptic glutamate release and enhanced post-synaptic A2AR-regulated AMPAR function that underlies APSPs [4, 5]. A previous study by Lopes et al. provides further support for a potential presynaptic mechanism involving a crosstalk between A1R and A2AR, in which it was shown that the facilitated hippocampal synaptic transmission observed with treatment with A2AR agonist was indirectly caused by attenuating the tonic effect of inhibitory presynaptic A1R [58].

Previous reports showed that prolonged A1R stimulation with A1R-selective agonist, during a 20-min hypoxia or after 48 h of pial vessel disruption (a focal cortical animal stroke model) led to clathrin-mediated internalization of GluA2 and GluA1 AMPARs, and this was also accompanied by decreased surface expression of A1Rs and increased surface expression of A2ARs in hippocampal slices [11, 12]. Within 5 min of A1R stimulation, peak levels of activated p38 MAPK, JNK, and protein phosphatase PP2A were observed and their levels recovered near baseline after 30 min [11, 38, 59]. These signaling proteins have also been implicated in internalization of epidermal growth factor, AMPARs and G protein coupled receptors [11, 12, 60–62]. Our present result with dynasore, a well-established small molecule inhibitor of dynamin-dependent and clathrin-mediated endocytosis, showed that APSPs were significantly reduced (albeit by only ~ 25%) but not abolished (i.e., fEPSPs did not recover back to baseline). Even higher concentrations of dynasore tested (i.e., 100 μM) did not produce a more profound inhibition of APSPs (unpublished). This suggested that a clathrin-independent endocytosis pathway may be involved in A1R or AMPAR endocytosis during the early (i.e., first 5 min) exposure to hypoxia. It is possible that the newly described clathrin-independent endocytic route known as fast endophilin-mediated endocytosis (FEME) [63] may also contribute to the generation of APSPs. Specifically, future studies are needed to address the relative contributions of clathrin-mediated and clathrin-independent internalization of A1Rs and AMPARs to APSP generation. However, it is worth noting that the YG peptide inhibitor of clathrin-mediated endocytosis of GluA2 AMPAR completely blocked all APSPs, indicating that GluA2 internalization triggers APSP induction.

In addition to this A1R-dependent, clathrin-mediated removal of GluA2 AMPARs from the surface membranes, the increased forward trafficking of A2AR and GluA1-containing AMPARs to nerve surface membranes after normoxic reperfusion [12] could also make a major contribution to APSP generation. However, the signaling pathways downstream of A1R signaling that could have a direct impact on A2AR signaling and APSP induction have yet to be identified. Previous reports have so far identified the protein kinase CK2 as a major regulator of Gαs-coupled GPCRs, with the CK2β subunit of the holoenzyme (comprised of 2 CK2α or CK2α’ and 2 CK2β subunits) interacting with Gαs or with the CK2α catalytic subunit phosphorylating an intracellular target substrate of the Gαs-coupled GPCRs which promotes receptor endocytosis or desensitization. Our results with the CK2 inhibitor DRB increasing A2AR surface expression and with the CK2 activator spermine reducing A2AR surface expression are entirely consistent with CK2 acting as a negative regulator of Gαs-coupled GPCRs [22, 23]. However, it is interesting that we observed the opposite effect of CK2 activity on the inhibitory Gαi-coupled A1Rs, namely increased A1R surface expression with spermine and decreased A1R surface expression with DRB. We observed hippocampal neuroprotection with DRB after hypoxia/reperfusion injury, which was also accompanied by profound reduction in A2AR surface expression. This observed neuroprotection could result from the decreased A2AR-mediated GluA1 surface insertion (i.e., decreased PKA phosphorylation of Ser845-GluA1), meaning decreased surface expression of CP-AMPARs. Alternatively, the DRB-induced neuroprotection could also result from decreased CK2-mediated phosphorylation of CP-AMPAR GluA1 subunits, as has been suggested in studies of cultured cortical neurons [64]. Inhibition of CK2 activity may also promote neuroprotection by inhibiting CK2 interaction with and subsequent activation of PP2A [27], and both of these proteins have been implicated in various neurodegenerative diseases [25, 65]. Since we observed increased A2AR surface expression during hypoxia/reperfusion, which correlated with increased hippocampal neuronal damage, and pretreatment with DRB dramatically reduced A2AR surface expression and hippocampal neuronal damage during hypoxia/reperfusion, together these results suggest that CK2 inhibition could represent a novel therapeutic target for stroke therapy.

AMPAR antagonists have already demonstrated neuroprotective effects in global cerebral stroke, focal cortical ischemia, spinal cord injury, and traumatic brain injury models [66] and have also been shown to have a longer therapeutic window compared to NMDAR antagonists of up to 6 h after the onset of ischemia [67, 68]. In our study, we showed that the NMDARs played little or no role in maintaining the APSPs in hippocampal CA3-CA1 synapses, but NMDARs contributed to post-hypoxia neuronal damage as they are expected to be under regulation by A2ARs to control neuronal damage, similar to other findings from the mossy fiber-CA3 pyramidal synapses [69]. We further explored the mechanism by which adenosine receptors regulate CP-AMPAR trafficking and neuronal damage. We showed that inhibition of CP-AMPARs with either IEM 1460 or Philanthotoxin-74 only exhibited a neuroprotective effect when administered early during hypoxic insult and present throughout normoxic reperfusion. Since we previously observed a rapid A1R-mediated, clathrin-dependent endocytosis of AMPARs during hypoxia [11, 16] and increased CP-AMPAR surface expression following hypoxia/normoxia reperfusion [12], it is plausible to suggest that a transient increase in CP-AMPAR surface expression contributes to APSP and neuronal damage. In contrast, the CP-AMPAR antagonists did not show significant neuroprotective effects when administered after the hypoxic insult. Interestingly, the well-tolerated and clinically approved AMPAR antagonist perampanel successfully showed neuroprotective effects when applied during both hypoxia and post-hypoxic conditions (early and late treatments). This ability of perampanel to exhibit neuroprotection in our ex vivo brain slice hypoxia/reperfusion injury model requires further investigation and specifically requires further elucidation of the precise binding site(s) of perampanel to GluA1 and GluA2 AMPAR subunits compared with other AMPAR antagonists [36, 70]. However, it is increasingly recognized that perampanel could have other non-glutamatergic receptor targets, including a recent finding showing perampanel-induced changes in the activity of PP2B, JNK, and other protein kinases in epilepsy [71]. While our data demonstrate that the reduction of APSP and neuronal death during post-hypoxia normoxic washout could be a consequence of the selective non-competitive binding of perampanel to AMPARs, they are not sufficient to rule out other pharmacological activity, such as the possible pharmacological effects on GABA-ergic systems as observed with CNQX [30] and on various signaling molecules [71]. CP-AMPARs are also suggested to be associated with other neurological disorders and neurodegenerative processes [72, 73], such as seizures [36] and Alzheimer’s and Parkinson’s diseases [5, 73]. The role of adenosine receptors in regulating CP-AMPAR surface expression and/or activation is an important area for future study, which may give further insight into how CP-AMPARs contribute to neuronal damage in these neurodegenerative disorders.

## Conclusion

**Fig. 9:**
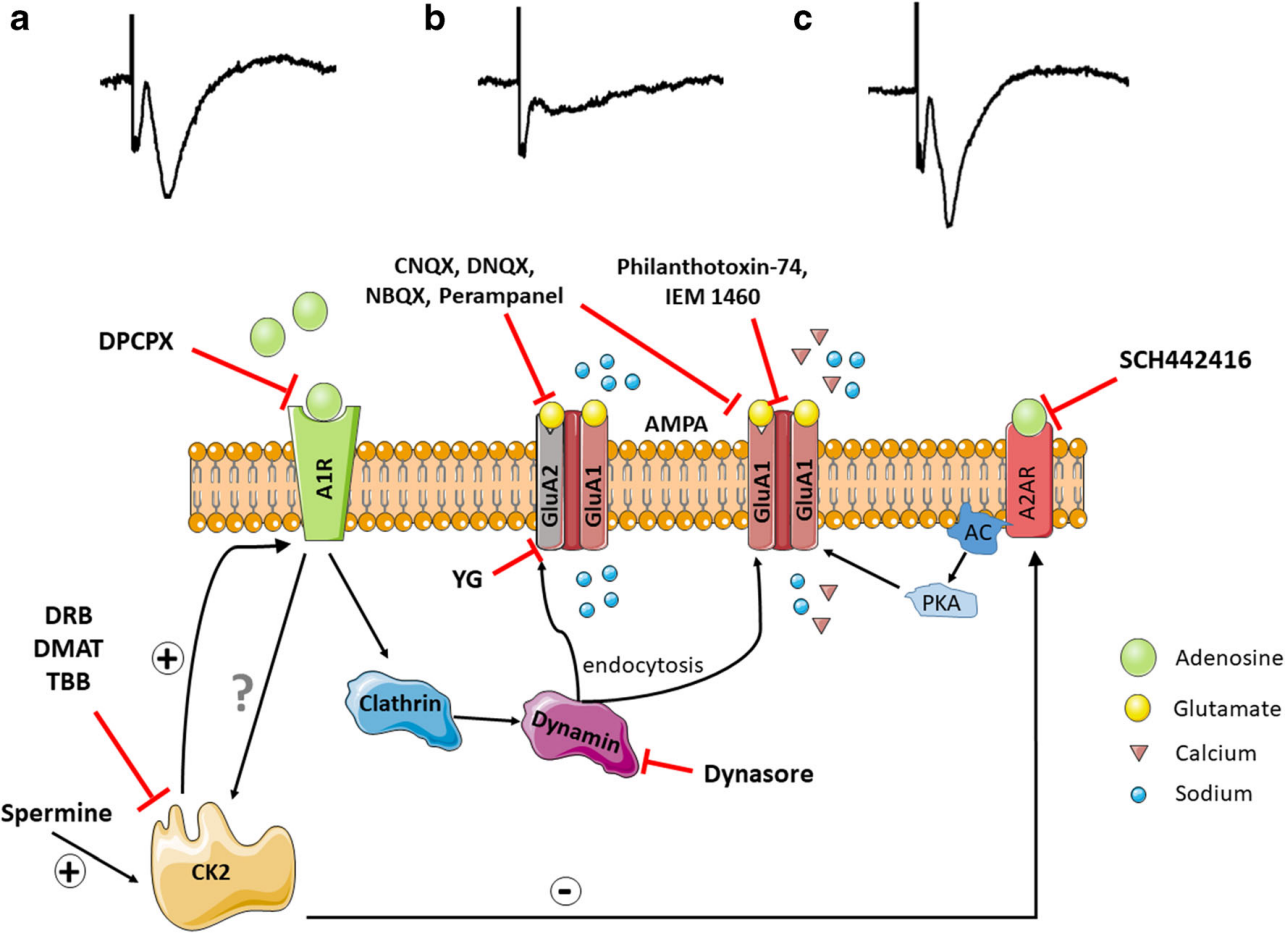
A proposed mechanism of post-hypoxia synaptic potentiation involving adenosine receptor crosstalk via CK2 and subsequent upregulation of calcium-permeable AMPARs. **a.** Normal condition: A2AR is negatively regulated by CK2, while the inhibitory A1R has the dominant effect in regulating AMPAR surface expression required for baseline synaptic transmission. **b.** Hypoxia: Hypoxia results in elevation of extracellular adenosine which mediates a temporary neuroprotective effect via A1R stimulation, manifested as synaptic depression. A1R stimulation inhibits presynaptic glutamate release and triggers dynamin-dependent clathrin-mediated endocytosis of GluA1 and GluA2 subunits of AMPAR. **c.** Normoxic reperfusion: Following the 20-min hypoxia, A1R is desensitized and A2AR surface expression is upregulated due to reduced CK2 function, and this leads to increased APSP. APSP is prevented by preincubation with either A1R or A2AR antagonist. Synaptic transmission during APSP is attenuated by calcium-permeable (CP)-AMPAR antagonists (IEM 1460, Philanthotoxin-74), or perampanel applied 5 min after hypoxia onset; only perampanel restores APSP to baseline levels if applied late during normoxic reperfusion. APSP is attenuated by CK2 or dynamin inhibitors and completely abolished by Tat-YG peptide inhibitor of clathrin-dependent endocytosis of AMPARs. The A1R-CK2-A2AR axis is a novel therapeutic target to prevent the AMPAR-mediated post-hypoxia synaptic potentiation and neurodegeneration

In this study, we demonstrated that a prior A1R-induced clathrin-mediated endocytosis of GluA2 AMPARs and a post-hypoxia A2AR-mediated enhancement of CP-AMPARs are reciprocally regulated by CK2, which leads to increased APSPs and hippocampal neuronal damage after hypoxia (see Fig. 9). We showed that AMPARs, and not NMDARs, are important in hippocampal CA1 neuronal excitability and play a crucial role in hypoxia-induced signaling and neuronal death. We also showed that APSP is attenuated with AMPAR antagonism and not with NMDAR antagonism. Unlike the NMDAR antagonists we tested, the pharmacological blockers of CP-AMPARs, IEM 1460 and philanthotoxin-74, were only effective in attenuating APSPs and neuronal damage when administered early after the onset of hypoxia. Perampanel, on the other hand, provided effective neuroprotection even after APSP induction, suggesting that this clinically relevant AMPAR antagonist may be repurposed for neuroprotective stroke therapy, perhaps offering a longer therapeutic window for treatment. We have also identified that CK2 inhibition could be an attractive neuroprotective therapy in stroke, but there are currently no clinically relevant drugs intended to function as CK2 inhibitors that can cross the blood–brain barrier. Therefore, our current study provides greater rationale for further drug development targeting the adenosinergic and glutamatergic signaling for future neuroprotective stroke therapy.

## Supplementary Information

ESM 1 (DOCX 1357 kb)

## Data Availability

All the data generated or analyzed during this study are included in this manuscript. Original raw data are available from the University of Saskatchewan (Department of Surgery) and can be readily furnished upon request.

## Abbreviations

A1R: Adenosine A1 receptor
A2AR: Adenosine A2A receptor
aCSF: Artificial cerebrospinal fluid
APSP: Adenosine-induced post-hypoxia synaptic potentiation
CK2: Casein kinase 2
CP-AMPAR: Ca^2+^-permeable AMPA receptor
fEPSP: Field excitatory post-synaptic potential
PVD: Pial vessel disruption
PI: Propidium iodide
