# Supplementary material for: Adenosine Signaling and Clathrin-Mediated Endocytosis of Glutamate AMPA Receptors in Delayed Hypoxic Injury in Rat Hippocampus: Role of Casein Kinase 2
Source: Mol Neurobiol. 2021 Jan 7;58(5):1932–51. doi: 10.1007/s12035-020-02246-0 (PMC8018935; doi:10.1007/s12035-020-02246-0)
Supplement: Supplementary file 1 — (DOCX 1357 kb) [file 12035_2020_2246_MOESM1_ESM.docx]

**Supplementary Figures**

**
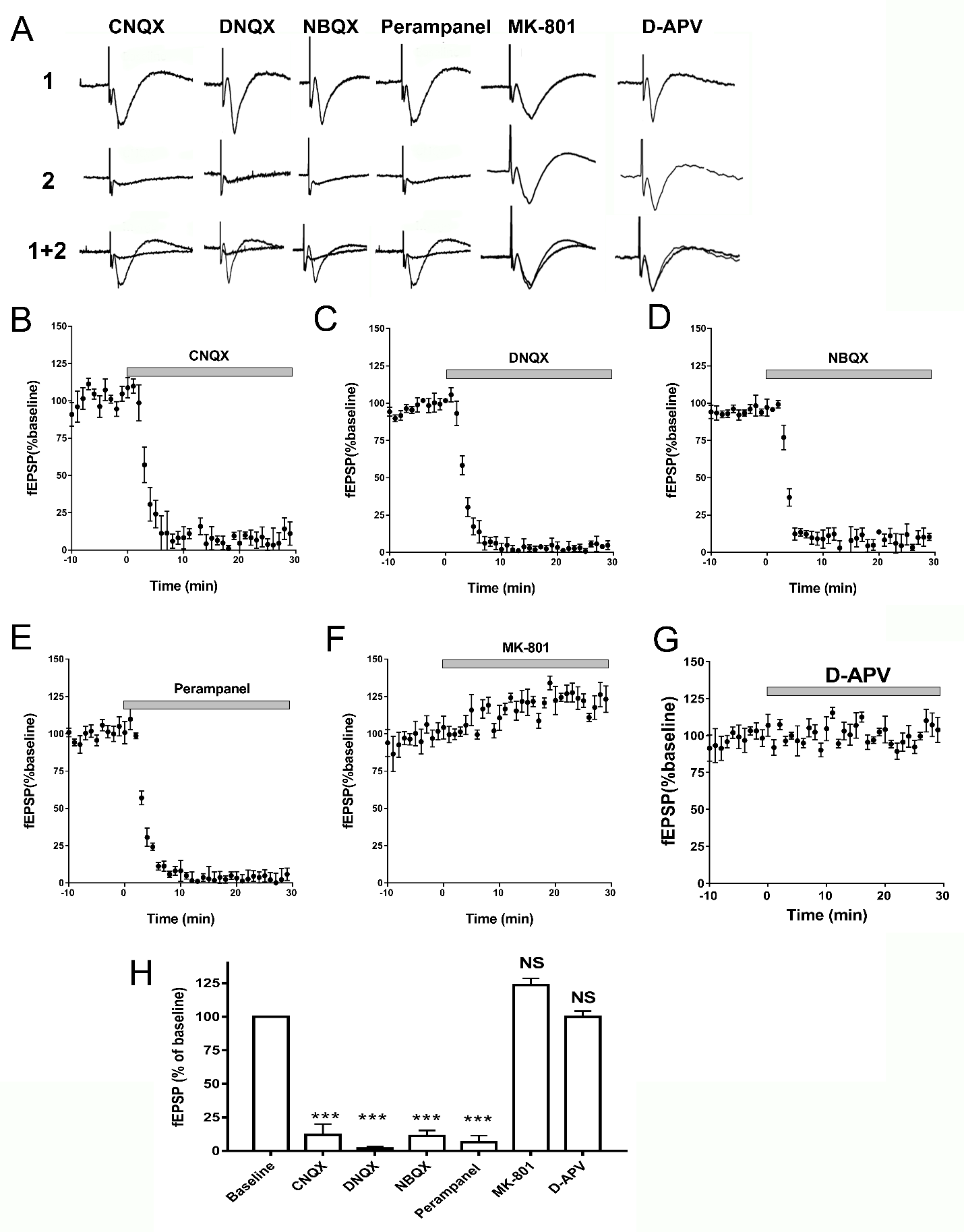
**

**Supplementary Figure 1: Synaptic transmission in CA1 layer of hippocampus is mainly mediated by AMPARs not NMDARs.** Following a 10 min baseline recording, hippocampal slices were treated with a selective AMPAR or NMDAR antagonist for 30 min, and all recordings were normalized to the last 5 min (10 sweeps) of the baseline (100%). A. Representative traces showing the average traces during the final 5 minutes of the baseline (1), the end of the 30 min drug treatment (2), and an overlay of both traces (1 + 2). Scale bars show 10 ms (x), 0.5 mV (y). Drug treatments were given at the following concentrations: CNQX (10 µM), DNQX (10 µM), NBQX (10 µM), Perampanel (10 µM), MK-801 (5 µM), and D-APV (100 µM). B-G. Time-course plot showing the mean fEPSP slope values as a percentage of the baseline (normalized to 100%) throughout the course of the experiment after perfusing AMPAR or NMDAR antagonists. H. Bar graph showing mean fEPSP slope values (% of baseline) of hippocampal slices treated with AMPAR, or NMDAR antagonists. All AMPARs used including CNQX, DNQX, NBQX and Perampanel significantly abolished fEPSPs; however, neither MK-801 nor D-APV caused significant changes in fEPSP values compared to baseline (100%). N = 7 independent experiments per treatment group. Significance: NS= non-significant, *** = p < 0.001.


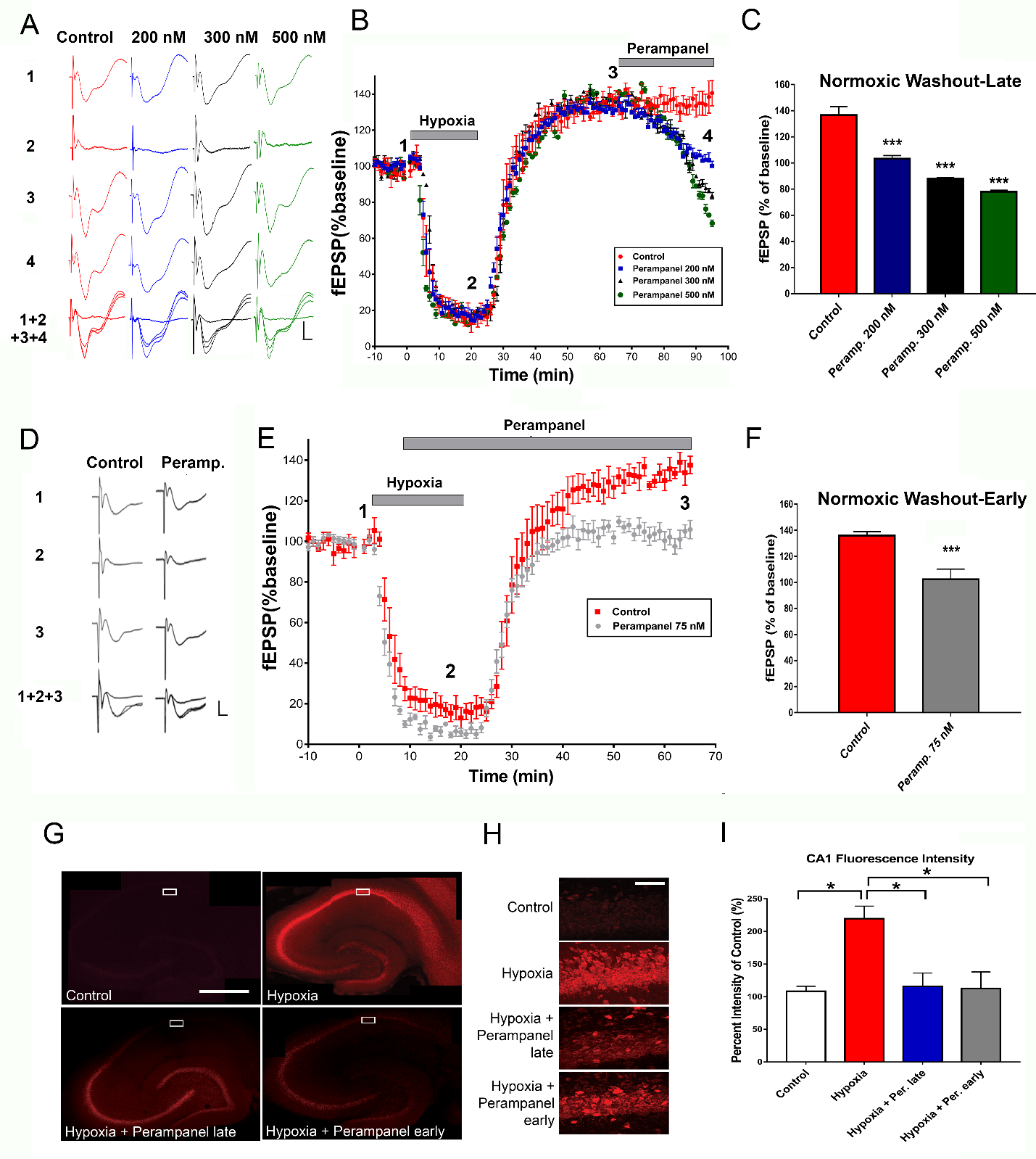


**Supplementary Figure 2. Perfusion of** **Perampanel following normoxic washout attenuated APSP in a concentration dependent manner.** A. Sample traces of the fEPSP experiments where Perampanel was given after the 45 min normoxic washout showing an average of the last 5 min of the 10 min baseline (1), 20 min hypoxia (2), 45 min normoxic washout (3), 30 min Perampanel treatment (200 nM in blue, 300 nM in black and 500 nM in green) (4), and an overlay of all traces (1+2+3+4). Scale bars show 10 ms (x), 0.5 mV (y). B. Time course graph showing normalized mean fEPSP both control (no Perampanel in red color) and slices treated with Perampanel late (200 nM in blue, 300 nM in black and 500 nM in green colors). C. Summary bar graph showing the average fEPSP value as a percentage of the baseline (100%) in the last 5 min of the 30 min Perampanel treatment period. Perampanel significantly reduced the APSP back to baseline values (200 nM) or below baseline values (300 nM and 500 nM), whereas control slices exhibited APSP. All graphed values showed Mean ± SEM. N = 6 independent fEPSP recordings per treatment group. Significance: *** = p<0.001.
